# *Trypanosoma cruzi*-specific IFN-γ-producing cells in chronic Chagas disease associate with a functional IL-7/IL-7R axis

**DOI:** 10.1371/journal.pntd.0006998

**Published:** 2018-12-05

**Authors:** María A. Natale, Gonzalo A. César, María G. Alvarez, Melisa D. Castro Eiro, Bruno Lococo, Graciela Bertocchi, María C. Albareda, Susana A. Laucella

**Affiliations:** 1 Instituto Nacional de Parasitología Dr. Fatala Chaben, Buenos Aires, Argentina; 2 Hospital Interzonal General de Agudos Eva Perón, Buenos Aires, Argentina; Instituto de Ciências Biológicas, Universidade Federal de Minas Gerais, BRAZIL

## Abstract

**Background:**

The severity of cardiac disease in chronic Chagas disease patients is associated with different features of T-cell exhaustion. Here, we assessed whether the ability of T cells to secrete IFN-γ in response to *T*. *cruzi* was linked to disruption in immune homeostasis and inflammation in patients with chronic Chagas disease.

**Methodology/Principal findings:**

PBMCs from chronic Chagas disease patients and uninfected controls were examined for frequencies of *T*. *cruzi*-responsive IFN-γ-producing cells by ELISPOT and cellular expression and function of IL-7R using flow cytometry. Serum levels of IL-7, IL-21, IL-27, soluble IL-7R, and inflammatory cytokines were also evaluated by ELISA or CBA techniques. Patients possessing *T*. *cruzi*-specific IFN-γ-producing cells (i.e. IFN-γ producers) had higher levels of memory T cells capable of modulating the alpha chain of IL-7R and an efficient response to IL-7 compared to that in patients lacking (i.e. IFN-γ nonproducers) parasite-specific T-cell responses. IFN-γ producers also showed low levels of soluble IL-7R, high basal expression of Bcl-2 in T cells and low basal frequencies of activated CD25^+^ T cells. Modulation of IL-7R was inversely associated with serum IL-6 levels and positively associated with serum IL-8 levels. Circulating IL-21 and IL-27 levels were not associated with the frequency of IFN-γ producing cells but were reduced in less severe clinical forms of the disease. In vitro stimulation of PBMCs with IL-7 or IL-27 enhanced IFN-γ production in IFN-γ producers but not in IFN-γ nonproducers.

**Conclusions/Significance:**

Alterations of the IL-7/IL-7R axis and in the levels of inflammatory cytokines were linked to impaired *T*. *cruzi*-specific IFN-γ production. These alterations might be responsible of the process of immune exhaustion observed in chronic Chagas disease.

## Author summary

Mechanisms of acquired immune response against *Trypanosoma cruzi* antigens include both humoral and cellular components that might be critical in a chronic infection. Through a vast number of studies, several groups have postulated that, similar to other chronic infections, T-cell responses in chronic *Trypanosoma cruzi* infection are driven to exhaustion. Alterations in T-cell signaling pathways have emerged as one of the mechanisms of immune exhaustion. Here, we investigated whether the ability of T cells to secrete IFN-γ in response to *T*. *cruzi* was linked to the expression and function of the IL-7 receptor and the cytokines involved in regulating this axis in patients with different clinical forms of chronic Chagas disease. This study showed that the ability of T cells to secrete IFN-γ in response to *T*. *cruzi* is linked to an efficient modulation and function of IL-7R and low levels of inflammatory cytokines. Low IFN-γ-ELISPOT responses could not be reverted by *in vitro* treatment with IL-7. These findings contribute to our understanding of the long-term consequences of *T*. *cruzi*-infection and might be useful to delineate novel therapeutic strategies.

## Introduction

Chagas disease is a major health problem in Latin America and an increasing threat in other countries that are non-endemic for *Trypanosoma cruzi* infection [1–4]. The relevance of T cell-mediated immunity in controlling *T*. *cruzi* infection has been demonstrated in human *T*. *cruzi* infections and in experimental models [5–9].

Individuals chronically infected with *T*. *cruzi*-have several indicators of T-cell exhaustion. A major finding was an overall low level of detectable *T*. *cruzi*-specific T cells and a predominance of single cytokine interferon (IFN)-γ only-producing T cells in the circulation of subjects with long-term *T*. *cruzi* infections [10–12]. Other feature of immune exhaustion of T cells in chronic Chagas disease is the expression of cytotoxic T lymphocyte antigen 4 by IFN-γ-producing CD4^+^ T cells in response to *T*. *cruzi* [13] and in the total T-cell compartment [13–15]. CTLA-4 expression was also observed in CD3^+^ T lymphocytes infiltrating the heart tissues of chronically infected subjects with severe myocarditis [13]. These findings suggest that parasite persistence induces overexpression of inhibitory receptors that might regulate deleterious consequences of a sustained immune response but also dampens the parasite-specific T-cell responses necessary for parasite control.

Interleukin-7 (IL-7) plays an important role in the maintenance of naïve and memory T cells by homeostatic mechanisms [16]. The IL-7 cell-surface receptor (IL-7R) comprises two chains, namely, the specific IL-7Rα (CD127) chain and the common γ-chain (CD132 or γc) [17]. The regulation of each chain is different; CD127 is downregulated, whereas the CD132 chain is rapidly upregulated upon T-cell activation [18]. Soluble IL-7R (sCD127) binds to IL-7 with an affinity similar to that of membrane-bound IL-7R [19], leading to sIL-7R-mediated inhibition of IL-7 signaling in T cells [20–21]. Inflammation perturbs the IL-7 axis, promoting senescence and exhaustion [22–23].

In a previous study, we found that chronic Chagas disease patients with severe cardiomyopathy have impaired function of IL-7R in total T cells [24]. Here, we sought to investigate whether the ability of T cells to produce IFN-γ in response to *T*. *cruzi* antigens was associated with the expression and function of IL-7R, with serum concentrations of the soluble form of IL-7R, STAT5 signaling, and inflammatory cytokines in chronic Chagas disease subjects with different degrees of cardiac dysfunction. In addition, *in vitro* treatment of PBMCs with IL-7 or IL-27 to enhance *T*. *cruzi*-specific IFN-γ production was evaluated. We showed that the ability of T cells to secrete IFN-γ in response to *T*. *cruzi* was associated with a functional IL-7/IL-7R signaling pathway in memory T cells, low levels of sCD127 and enhanced basal expression of Bcl-2 on T cells.

## Materials and methods

### Ethics statement

This study was approved by the Institutional Review Board of the Hospital Interzonal General de Agudos Eva Perón. All patients signed informed consent forms prior to inclusion in the study.

### Selection of study population

*T*. *cruzi*-infected subjects were recruited at the Chagas Disease Unit Cardiology Department, Hospital Interzonal General de Agudos Eva Perón, Buenos Aires, Argentina. *T*. *cruzi* infection was determined with indirect immunofluorescence assays, hemagglutination and ELISA tests [25]. Patients positive in at least two of these tests were considered to be infected. Subjects were clinically evaluated and grouped according to a modified version of the Kuschnir grading system [26]: Group 0 (G0), seropositive individuals exhibiting a normal electrocardiogram (ECG) and normal echocardiograph; Group 1 (G1), seropositive individuals with a normal echocardiograph but ECG abnormalities; Group 2 (G2), seropositive individuals with ECG abnormalities and heart enlargement; and Group 3 (G3), seropositive individuals with ECG abnormalities, heart enlargement and clinical or radiological evidence of heart failure. These individuals were originally infected while living in areas where *T*. *cruzi* infection was endemic but had lived in an area where *T*. *cruzi* infection was not endemic for an average of 30 years. Two patients both in the G2 clinical stages had been treated with benznidazole six years prior to inclusion in this study. The serologic titers against *T*. *cruzi* did not decrease after treatment and in one of them the clinical stage changed from G1 to G2 after benznidazole administration, supporting that treatment was not successful in these two patients. Healthy subjects from Buenos Aires that have always resided in non-endemic areas and with negative serology for *T*. *cruzi* infection served as the uninfected group (UI). The primary characteristics of the study population are summarized in Table 1. *T*. *cruzi*-infected patients and uninfected controls with hypertension, ischemic heart disease, cancer, HIV infection, syphilis, diabetes, arthritis or serious allergies were excluded from this study.

**Table 1:** Characteristics of study population.

Patient group	n	Age range	Residency in	Etiological	Sex	
			non-endemic			
		(median),	areas range			
		years	(median), years	treatment	Male	Female
G0	46	26–63 (46)	10–53 (30)	0/46	19	27
G1	28	21–66 (46)	3–50 (34)	0/28	12	16
G2	13	26–64 (48)	7–63 (26)	2/13	8	5
G3	16	40–76 (58)	15–65 (43)	0/16	12	4
Uninfected	37	28–65 (45)	28–65 (45)	0/37	14	23

### Collection of peripheral blood mononuclear cells (PBMCs) and sera

Approximately 50 mL of blood was drawn by venipuncture into heparinized tubes (Vacutainer, BD Biosciences). PBMCs were isolated by density gradient centrifugation with Ficoll-Hypaque medium (Amersham) and resuspended in a volume of RPMI 1640 medium (Corning) supplemented with 10% heat-inactivated FBS (NOTACOR). Cells were then cryopreserved with an equal volume of freezing media containing 20% DMSO and 80% FBS and stored in liquid nitrogen until use. For serum separation, blood was allowed to coagulate at room temperature and centrifuged at 2000 rpm for 10 min. Then, serum was aliquoted and stored at -70°C until use. Cell viability was evaluated by trypan blue staining (80–95% viable cells/sample) prior to use. Due to sample availability, the assays were not run for all samples.

### *Trypanosoma cruzi* antigens

Protein lysate from *T*. *cruzi* amastigotes derived from the Brazil strain was obtained by four freeze/thaw cycles followed by sonication, as previously reported [10].

### IFN-γ enzyme-linked immunosorbent spot (ELISPOT) assays

The number of *T*. *cruzi*-specific IFN-γ-producing T-cells was determined by *ex vivo* ELISPOT assays with a commercial kit (BD Biosciences), as described elsewhere [10]. PBMCs were stimulated with 10 μg/mL of a *T*. *cruzi* lysate preparation, with or without IL-7 (Abcam) or IL-27 (R&D), at 50 ng/mL final concentration. Stimulation with 20 ng/mL of PMA (Sigma) plus 500 ng/mL ionomycin (Sigma) or medium alone, with or without cytokines, was performed as a positive or negative control, respectively. The number of specific IFN-γ-secreting cells was calculated by subtracting the value of the wells containing media alone. Responses were considered positive when a minimum of 10 spots/4×10^5^ PBMCs were present per well, and this number was at least twice the number present in wells with medium alone. Thereafter, subjects who showed positive IFN-γ ELISPOT responses were referred as “IFN-γ producers” and those with ELISPOT responses below background levels were referred as “IFN-γ nonproducers”.

### Monoclonal antibodies, gating strategies and data acquisition

Fluorescein (FITC)-conjugated anti-CD25 (catalog number 555431), phycoerythrin (PE)-conjugated anti-CD132 (catalog number 555900), allophycocyanin (APC), peridinin-chlorophyll proteins (PerCP)- and Pacific Blue (PB)-conjugated anti-CD4 (catalog numbers 555349, 347324 and 558116, respectively), PerCP- or FITC-conjugated anti-CD8 (catalog numbers 347314 and 555634, respectively), Alexa Fluor 647-conjugated anti-CD127 (catalog number 558598), FITC-conjugated anti-CD45RA (catalog number 555488), PE-conjugated anti-phosphorylated STAT5 (612567), PE Cy7-conjugated anti-PD-1 (catalog number 561272), Alexa Fluor 488-conjugated anti-IFN-γ (catalog number 557718) and Fixable Viability 510 (564406) were purchased from BD Biosciences. PE-conjugated anti-Bcl-2 (MHBCL04) was purchased from Thermo Fisher Scientific. Cell samples were acquired on a FACS Aria II flow cytometer (BD, USA) and analyzed with FlowJo software (Tree Star, San Carlos, CA, USA). Lymphocytes were gated based on their forward scattering and side scattering parameters, followed by the use of forward scatter area vs. forward scatter height dot-plot for doublet discrimination. The subsequent analyses were performed on viable cells (FV510^—^) (S1A Fig)

### Surface expression of IL-7R components in effector and memory T cells

One million PBMCs were stained with Fixable Viability 510 (FV510) according to the manufacturer’s instructions. Then, these PBMCs were stained with anti-CD4 PerCP, anti-CD8 PerCP, anti-CD45RA FITC, anti-CD127 Alexa Fluor 647 and anti-CD132 PE for 30 min on ice. Then, cells were washed and resuspended in PBS containing 2% paraformaldehyde (PFA). Memory and effector T cells were gated according to CD45RA and CD127 expression in CD4^+^ and CD8^+^ T cells (S1A Fig).

### Intracellular STAT5 phosphorylation assay and evaluation of Bcl-2 and CD25 expression following *in vitro* IL-7 stimulation

IL-7-induced STAT5 phosphorylation, as well as Bcl-2 and CD25 expression in PBMCs, was determined as previously described [24]. Briefly, 2x10^6^ PBMCs were cultured overnight in serum-free medium (AIM-V, Invitrogen, Carlsbad, USA) followed by a 15 min incubation with 100 ng/mL recombinant human IL-7 (rhIL-7, Abcam) for the STAT5 phosphorylation assay or cultured for two days in complete RPMI medium with or without 10 ng/mL of rhIL-7 for Bcl-2 and CD25 expression analysis at 37°C, 5% CO_2._ Then, cells were labeled with anti-CD4 APC, anti-CD8 PerCP/FITC or anti-CD25 FITC on ice and immediately fixed. Cells were permeabilized, and intracellular staining with anti-phosphorylated STAT5 (pSTAT5) PE or anti-Bcl-2 PE was performed. IL-7-induced STAT5 phosphorylation (Δ % pSTAT5^+^) for CD4^+^ and CD8^+^ T cells was determined by calculating the difference in percentages of pSTAT5^+^ cells between IL-7-stimulated and unstimulated samples. Since Bcl-2 is constitutively expressed, the change in the mean fluorescence intensity (MFI) was used to evaluate induction above basal levels. The induction of Bcl-2 and CD25 expression was measured by subtracting the MFI for Bcl-2, or the percentages of CD25-expressing T cells in unstimulated cultures, from those in IL-7-stimulated cultures.

### Intracellular IFN-γ staining assays

For sixteen to twenty hours, 4×10^6^ PBMCs were incubated with 15 μg/mL of lysate preparation [10] or media alone plus 1 μg/mL CD28/CD49d (BD Biosciences) at 37°C in a CO_2_ incubator. Brefeldin A (10 μg/mL; Sigma) was added for the last 5 h of incubation, as previously described [11, 12]. Stimulation with Staphylococcal enterotoxin B (SEB) (1 μg/mL; Sigma Aldrich) served as a positive control. Cells were stained with FV510 and anti-CD4 PB, anti-CD127 Alexa Fluor 647, anti-CD132 PE and anti-PD-1 PE-Cy7 monoclonal antibodies (BD Bioscience) for 30 min on ice followed by fixation and permeabilization for intracellular staining with anti-IFN-γ (AF488) (BD, Bioscience). CD127, CD132 and PD-1 expression levels were quantified in IFN-γ-producing and non-IFN-γ-producing CD4^+^ T cells.

### Measurement of cytokines and sCD127 in serum samples

Serum levels of IL-7, IL-9, IL-21and IL-27(Abcam)and sCD127 (MyBioSource) were measured in duplicate using ELISA kits. Inflammatory cytokines including IL-1β, IL-6, IL-8, IL-9, IL-10, IL-12 and TNF-α, were measured by Cytometric Bead Array (CBA, BD Biosciences) according to the manufacturer’s instructions.

### *In vitro* expansion of *T*. *cruzi*-specific T cells

Cells (3×10^6^) were plated in 24-well cell-culture plates in a total volume of 1.5 mL complete RPMI with 10% FBS and incubated for 10 days at 37°C, with 5% CO_2_ atmosphere, 99% humidity, along with *T*. *cruzi* lysate (10 μg/mL final concentration), in the presence or absence of rhIL-7 or rhIL-27 (25 ng/mL final concentration). On day three, 20 IU/mL of IL-2 (BioLegend) was added to each well. After 10 days, PBMCs were harvested, washed, and resuspended in complete RPMI medium. Cultured PBMCs were tested for the presence of IFN-γ-secreting T cells in response to *T*. *cruzi* lysate using ELISPOT assay, by seeding 2×10^5^ cultured PBMCs/well along with 1×10^5^ autologous unstimulated cryopreserved PBMCs/well as antigen presenting cells.

### Statistical analysis

The normality of data was evaluated by the Shapiro-Wilk test. The results are given as medians and interquartile ranges. Differences between IFN-γ producers and IFN-γ nonproducers of each clinical group and the uninfected group were determined by analysis of variance (ANOVA) followed by Bonferroni’s or Dunn’s multiple comparisons test, as appropriate according to the normality of data, or by a test for lineal trend. The correlations between variables was determined by a Spearman or Pearson test, as appropriate and were considered significant when p≤0.05. Univariate analysis defining the ability to secrete IFN-γ in response to *T*. *cruzi* antigen stimulation as the outcome was evaluated with the Wilcoxon rank sum test or the two-sample *t*-test, as appropriate, for continuous variables. All parameters in the univariate analysis with a p<0.05 and two variables with p<0.1 were transformed into log scale. Correlation analyses between variables were performed to incorporate each variable into one out of six logistic regression models for the multivariate analysis. We used odds ratios with 95% confidence intervals for the logistic regression analysis.

## Results

### Lack of IFN-γ-producing cells in response to *T*. *cruzi* was associated with impaired modulation of IL-7 receptor components in memory CD4^+^ and CD8^+^ T cells

**Fig 1:**
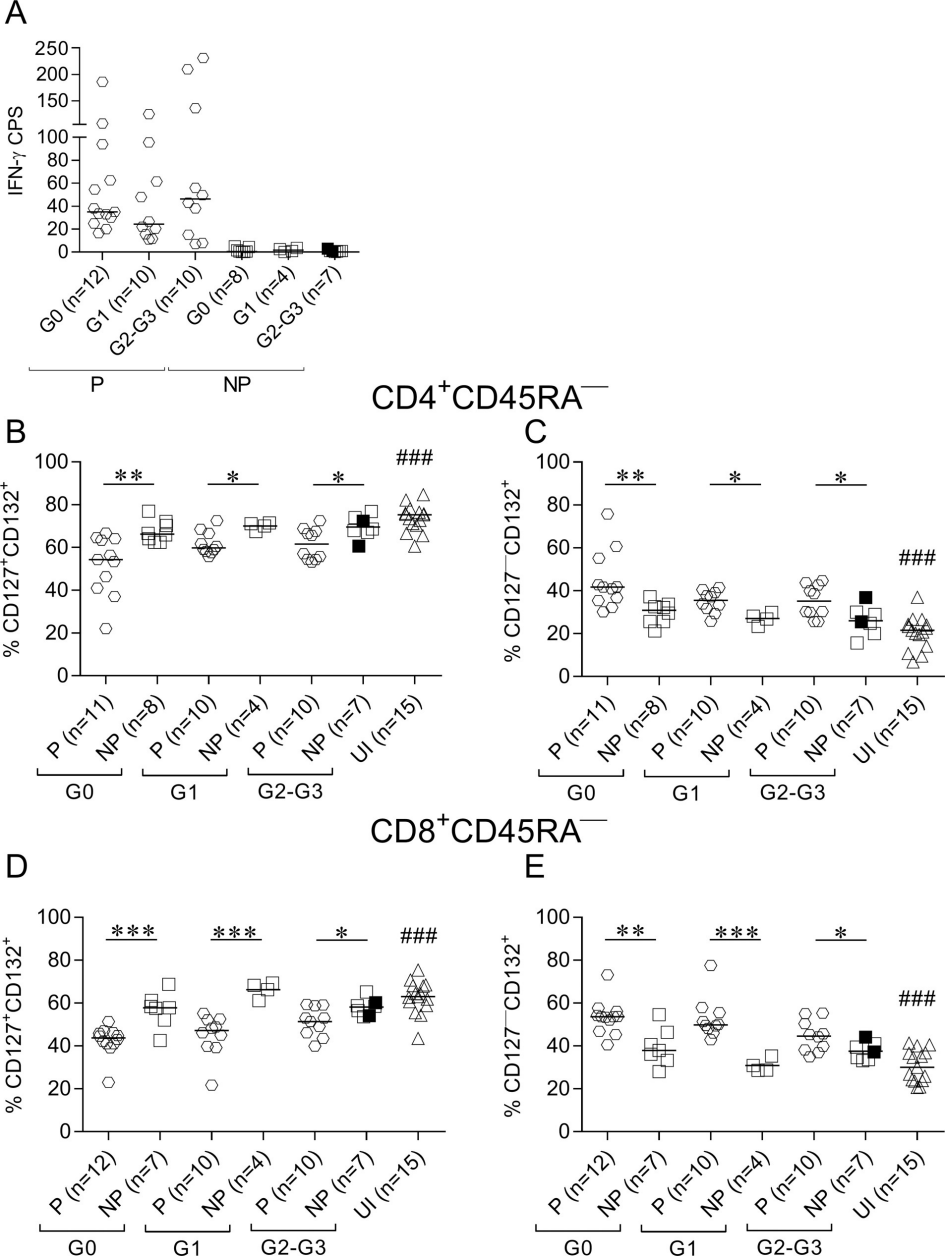
CD127 cell-surface expression is downregulated in memory CD4^+^ and CD8^+^ T cells in IFN-γ producers. PBMCs were stained with FV510, CD45RA, CD8, CD4, CD127, and CD132 monoclonal antibodies and analyzed using flow cytometry. *T*. *cruzi-*specific T-cell responses were determined using IFN-γ ELISPOT after stimulation of PBMCs with a *T*. *cruzi* lysate. Each symbol represents the number of *T*. *cruzi*-specific IFN-γ cells producing spots (CPS) by subtracting the value of wells containing media alone (A) and the proportion of CD127^+/—^CD132^+^ cells among total CD4^+^CD45RA^—^(B and C) or CD8^+^CD45RA^—^(D and E) T-cell populations. Median values are indicated as horizontal lines. Black symbols indicate the subjects treated with benznidazole. The responses of *T*. *cruzi*-infected subjects were used to determine the IFN-γ producers (P) and IFN-γ nonproducers (NP) based on the ELISPOT assay, as described in Materials and Methods. Comparisons between P and NP for each clinical group and uninfected subjects were performed using ANOVA followed by Bonferroni’s multiple comparisons test. * p ≤ 0.05, ** p ≤ 0.01, *** p ≤ 0.001. ### p ≤ 0.001 compared to P G0, P G1 and P G2-G3.

Subjects with measurable IFN-γ-producing T cells in response to *T*. *cruzi* lysate (i.e., IFN-γ producers, Fig 1A) exhibited lower frequencies of CD127^+^CD132^+^ cells and higher frequencies of CD127^—^CD132^+^ in CD4^+^ cells (Fig 1B and 1C; S1C Fig) and CD8^+^ (Fig 1D and 1E, S1D Fig) memory (CD45RA^—^) T cells compared with non-IFN-γ-producers and uninfected subjects. Memory CD4^+^ and CD8^+^ T cells with downregulated CD127 diminished with the intensification of disease severity in IFN-γ producers, and CD127^+^CD132^+^ increased (S2 Fig, test for linear trend between medians).

**Fig 2:**
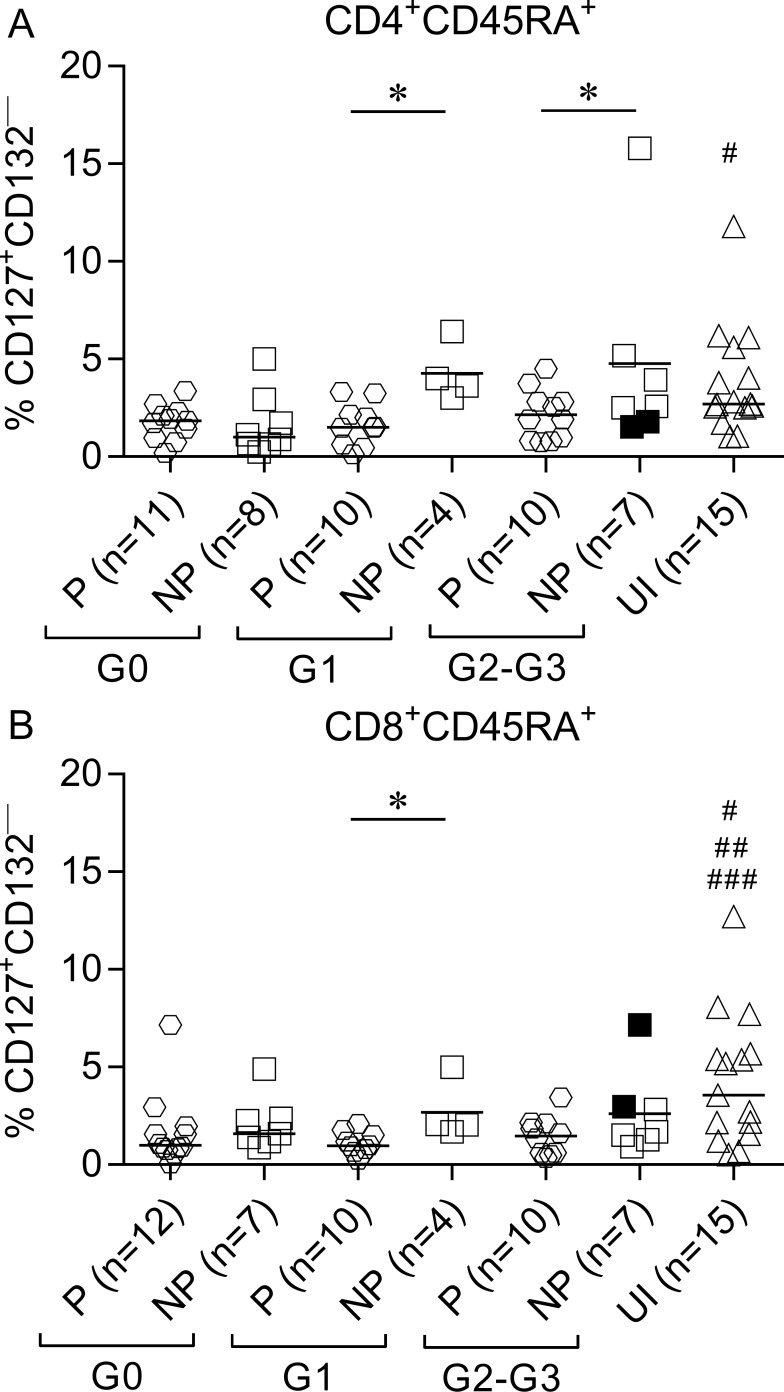
Decreased frequency of recent thymic emigrants in IFN-γ producers. PBMCs were stained with FV510, CD45RA, CD8, CD4, CD127, and CD132 monoclonal antibodies and analyzed using flow cytometry. Each symbol represents the proportion of CD4^+^CD45RA^+^CD127^+^CD132^—^(A) or CD8^+^CD45RA^+^CD127^+^CD132^—^(B) T-cell populations from IFN-γ producers (P) and IFN-γ nonproducers (NP), as classified according to ELISPOT responses as defined in Materials and Methods. Median values are indicated as horizontal lines. Black symbols indicate subjects treated with benznidazole. Comparison between P and NP for each clinical group and uninfected subjects were performed using ANOVA followed by Bonferroni’s multiple comparisons test. * p ≤ 0.05. (A) # p ≤ 0.05 compared with P G0, NP G0, P G1 and P G2-G3; (B) # p ≤ 0.05 compared with P G2-G3; ## p ≤ 0.01 compared with P G0; ### p ≤ 0.01 compared with P G1.

**Fig 3:**
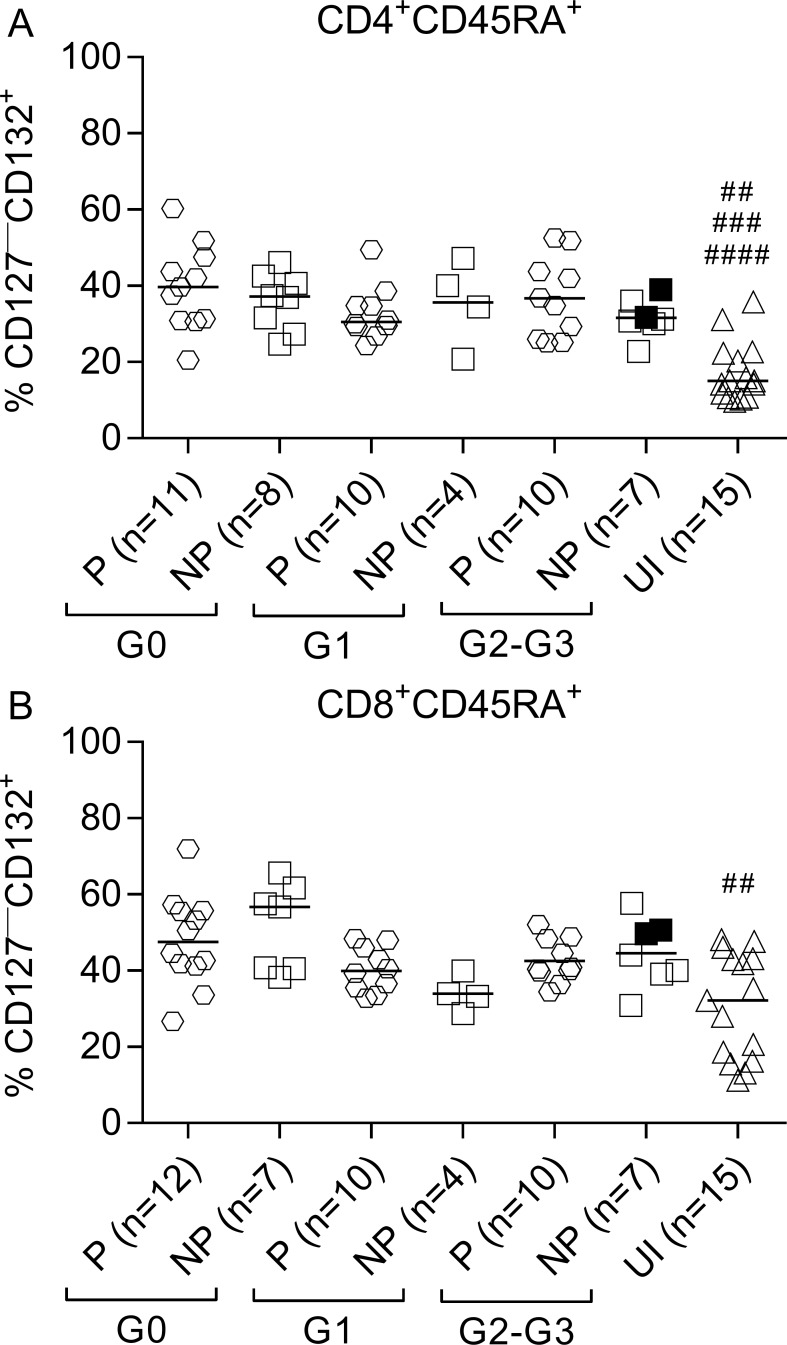
Increased frequency of TTE cells in patients with chronic Chagas disease. PBMCs were stained with FV510, CD45RA, CD8, CD4, CD127, and CD132 monoclonal antibodies and analyzed using flow cytometry. Each symbol represents the proportion of CD127^—^CD132^+^ cells in total CD4^+^CD45RA^+^ (A) or CD8^+^CD45RA^+^ (B) T cell populations from IFN-γ producers (P) and IFN-γ nonproducers (NP), as classified according to ELISPOT responses as defined in Materials and Methods. Median values are indicated as horizontal lines. Black symbols indicate subjects treated with benznidazole. Comparisons between P and NP for each clinical group and uninfected subjects were performed using ANOVA followed by Dunn’s multiple comparisons test. (A) ## p ≤ 0.01 compared to NP G1 and NP G2-G3; ### p ≤ 0.001 compared with P G1 and NP G0; #### p ≤ 0.0001 compared with P G0 and P G2-G3; (B) ## p ≤ 0.001 compared with P G0 and NP G0.

CD45RA^+^ T cells are primarily comprised of naïve and terminally differentiated effector T cells [27, 28]. We measured the proportion of recent thymic emigrant cells (RTE) (i.e., CD127^+^CD132^—^) and terminally differentiated T cells (TTE) (i.e., CD127^—^CD132^+^) [29–30] among CD45RA^+^ T cells based on the expression of CD127 and CD132. IFN-γ producers in patients with cardiac disease (i.e., subjects in the G1, G2 and G3 clinical groups) exhibited lower frequencies of RTE in the CD4^+^ and CD8^+^ T-cell compartments compared with IFN-γ nonproducers and uninfected subjects (Fig 2A and 2B). IFN-γ producers and IFN-γ nonproducers in G0 patients exhibited equally decreased values of CD4^+^ RTE compared to uninfected subjects (Fig 2A). In contrast, CD4^+^CD45RA^+^cells in *T*. *cruzi*-infected subjects were enriched in the TTE cells compared with uninfected subjects, regardless of the ability of T cells to respond to *T*. *cruzi* antigens and the clinical status (Fig 3A and S1E Fig). Only IFN-γ producers and IFN-γ nonproducers with no signs of cardiac disease exhibited equally significantly increased levels of TTE cells among CD8^+^ T cells (Fig 3B) compared with uninfected subjects. TTE levels were also slightly increased in patients with cardiac disease (Fig 3B).

### Higher frequencies of T cells with downregulated CD127 expression and PD-1 expression in IFN-γ-producing T cells in response to *T*. *cruzi*

**Fig 4:**
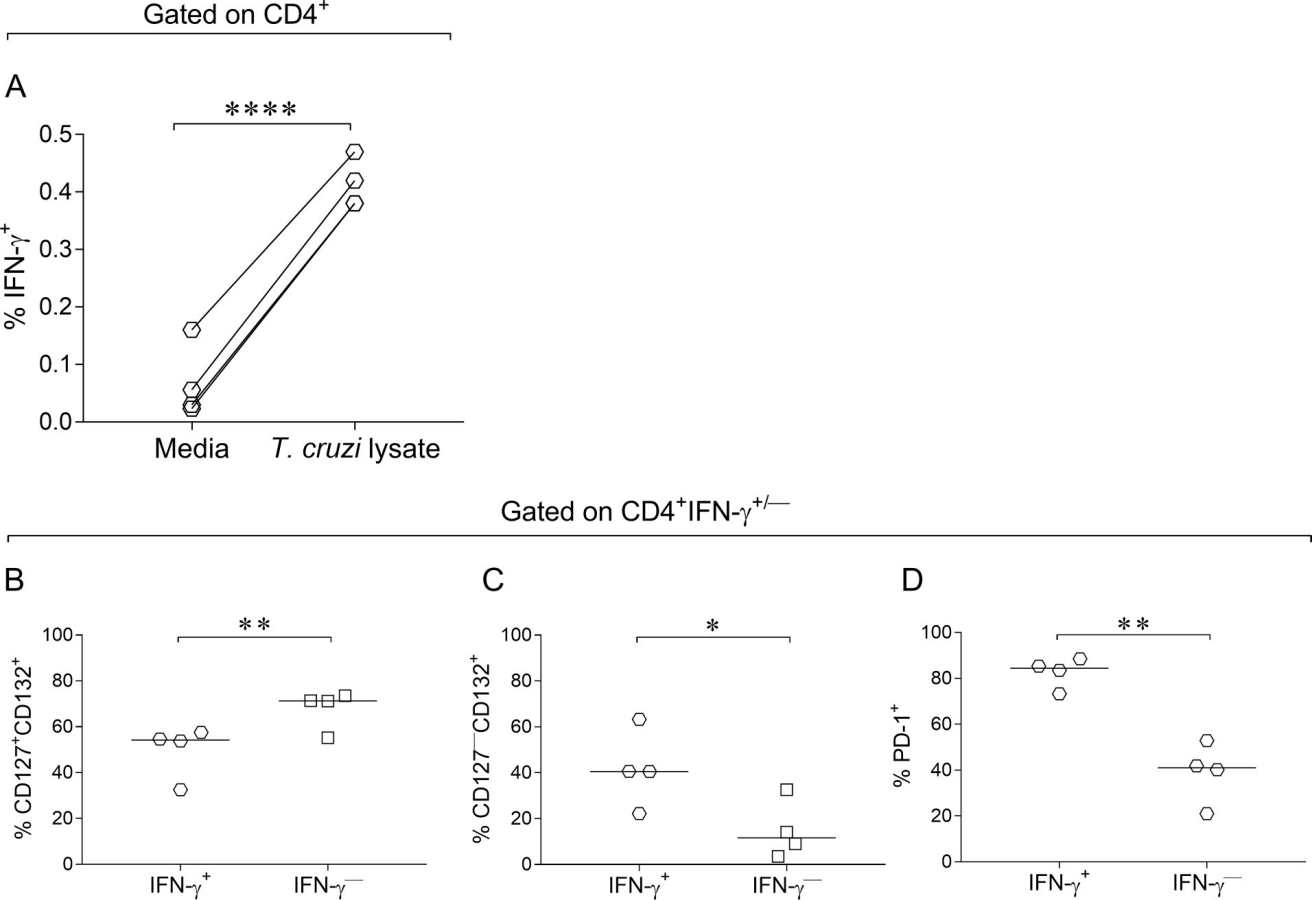
Increased downregulation of CD127 expression and upregulation of PD-1 expression in IFN-γ-producing CD4^+^ T cells. PBMCs were stimulated for 18–20 h with *T*. *cruzi* lysate or media alone. Cells were stained with FV510, CD4, CD127, CD132 and CD279 (PD-1) monoclonal antibodies followed by fixation and permeabilization for intracellular staining with anti-IFN-γ monoclonal antibody. Each symbol represents the expression of CD127^+/—^CD132^+^(A-C), or PD-1 (D) in IFN-γ-producing and IFN-γ nonproducing CD4^+^ T cells (D). Median values are indicated as horizontal lines. Comparisons between IFN-γ-producing and IFN-γ nonproducing groups were performed using paired *t* test. * p ≤ 0.05 and ** p ≤ 0.01 compared with IFN-γ nonproducing T cells. **** p ≤ 0.001 compared with media.

We examined whether IFN-γ-producing T cells in response to *T*. *cruzi* exhibited distinct expression of CD127 and CD132 chains of the IL-7R and the inhibitory receptor PD-1 compared with IFN-γ nonproducing T cells. IFN-γ-producing CD4^+^ T cells in response to a *T*. *cruzi* lysate were enriched in CD127^—^CD132^+^ T cells compared with IFN-γ nonproducing CD4^+^ T cells (Fig 4A–4C, S3 Fig). IFN-γ producing CD4^+^ T cells exhibited increased PD-1 expression compared with IFN-γ nonproducing cells (Fig 4D, S3 Fig).

### Impaired IFN-γ production is associated with increased basal levels of STAT5 phosphorylation and CD25 expression and decreased basal expression of Bcl-2 in T cells

**Fig 5:**
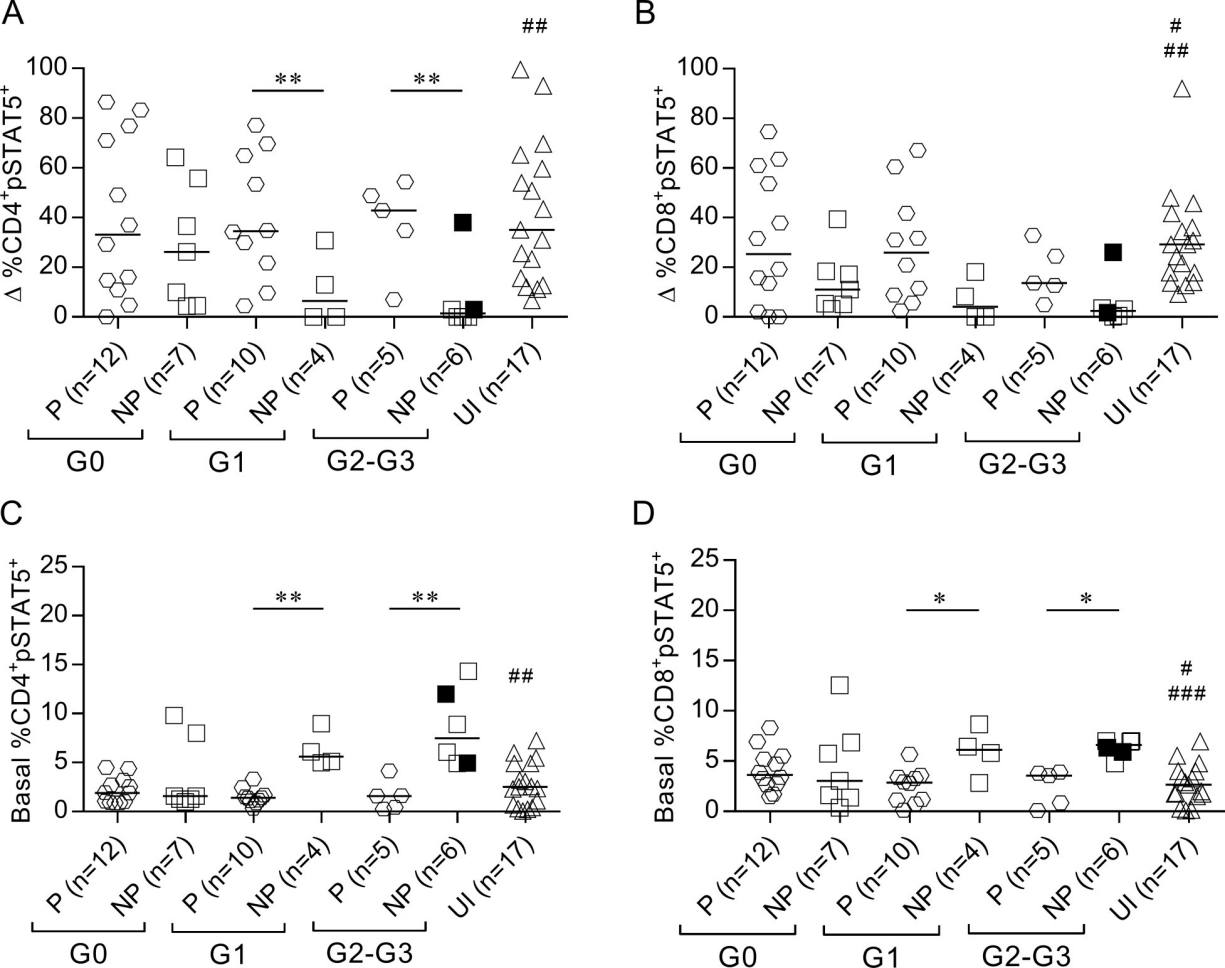
Altered STAT5 phosphorylation in IFN-γ nonproducers. pSTAT5 frequency was evaluated after stimulation with rhIL-7 in total CD4^+^ (left) and CD8^+^ (right) T cell populations using flow cytometry. Each point represents the difference (Δ) in the percentage of pSTAT5 expression between rhIL-7-stimulated and unstimulated samples (A-B) or basal frequency of pSTAT5^+^ (C-D). Horizontal lines indicate median values. Black symbols indicate subjects treated with benznidazole. Based on the ELISPOT assay, responses of *T*. *cruzi*-infected subjects were used to determine the IFN-γ producers (P) or IFN-γ nonproducers (NP), as described in Materials and Methods. Comparisons between P and NP for each clinical group and uninfected subjects were performed using ANOVA followed by Dunn’s multiple comparisons test. * p ≤ 0.05, ** p ≤ 0.01. (A) ## p ≤ 0.01 compared with NP G1 and NP G2-G3; (B) # p ≤ 0.05 compared with NP G0, ## p ≤ 0.01 compared with NP G2-G3 and NP G1; (C) ## p ≤ 0.01 compared with NP G1 and NP G2-G3; (D) # p ≤ 0.05 compared with NP G1, ### p ≤ 0.001 compared with NP G2-G3.

**Fig 6:**
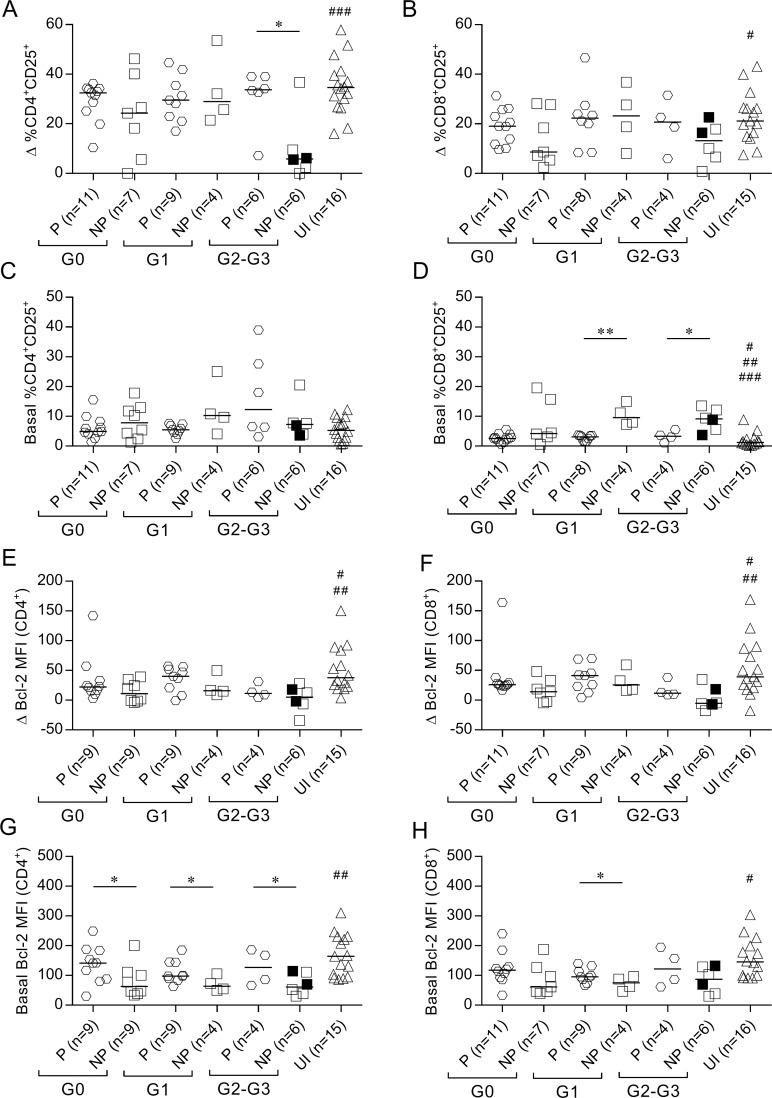
Altered STAT5 downstream events in IFN-γ nonproducers. Bcl-2 and CD25 expression was determined using flow cytometry analysis after 48 h of *in vitro* stimulation with rhIL-7. Each symbol represents the IL-7-induced CD25 expression after (Δ % CD25^+^) (A-B) or the basal CD25^+^ expression (C-D). The difference in Bcl-2 mean fluorescence intensity (MFI) between IL-7-stimulated and unstimulated cell cultures (E-F) or the basal Bcl-2 expression (G-H) in CD4^+^ (left) or CD8^+^ (right) T cells. Horizontal lines indicate median values. Black symbols indicate subjects treated with benznidazole. Based on the ELISPOT assay, responses of *T*. *cruzi*-infected subjects were used to determine the IFN-γ producers (P) and IFN-γ nonproducers (NP), as described in Materials and Methods. Comparisons between P and NP for each clinical group and uninfected subjects were performed using ANOVA followed by Dunn’s multiple comparisons test. * p ≤ 0.05, ** p ≤ 0.01. (A) ### p ≤ 0.001 compared with NP G2-G3; (B) # p ≤ 0.05 compared with NP G2-G3; (D) # p ≤ 0.05 compared with NP G0, ## p ≤ 0.01 compared with NP G1, ### p ≤ 0.001 compared with NP G2-G3; (E, F) # p ≤ 0.05 compared with NP G0 and NP G1, ## p ≤ 0.01 compared with NP G2-G3; (G) ## p ≤ 0.01 compared with NP G0, NP G1 and NP G2-G3; (H) # p ≤ 0.05 compared with NP G0, NP G1, NP G2-G3.

We evaluated the association between IFN-γ secretion capacity in response to *T*. *cruzi* and the function of IL-7 receptor as measured using STAT5 phosphorylation and CD25 and Bcl-2 expression in T cells following stimulation with rhIL-7. IFN-γ nonproducers with cardiac disease (i.e., subjects of the G1, G2 and G3 clinical groups) exhibited lower frequencies of phosphorylated STAT5^+^ (pSTAT5) in CD4^+^ T cells than IFN-γ producers and uninfected subjects, in response to IL-7 (Fig 5A, S4A and S4B Fig). CD8^+^pSTAT5^+^ T cells were also lower in IFN-γ nonproducers irrespective of clinical status, but this difference was not statistically significant (Fig 5B, S4C and S4D Fig). The lower functional capacity of the IL-7R in T cells of IFN-γ nonproducers in response to IL-7 was associated with increased basal levels of pSTAT5 (Fig 5C and 5D, S4B and S4D Fig) compared with IFN-γ producers and uninfected subjects. Decreased CD25 upregulation in response to IL-7 in CD4^+^ T cells was observed in IFN-γ nonproducers with severe cardiomyopathy compared with IFN-γ producers and uninfected subjects (Fig 6A). Most patients exhibited unaltered basal CD25 expression in T CD4^+^ T cells (Fig 6C). The basal expression of the activation marker CD25 in CD8^+^ T cells was increased in IFN-γ nonproducers with cardiac disease compared with IFN-γ producers and uninfected subjects (Fig 6D), but the upregulation of CD25 after IL-7 stimulation was not drastically impaired in subjects who lacked IFN-γ-producing cells (Fig 6B). A slight decrease in Bcl-2 expression in response to IL-7 along with decreased basal Bcl-2 expression levels was found in CD4^+^ and CD8^+^ T cells of IFN-γ nonproducers compared with IFN-γ producers and uninfected subjects (Fig 6E–6H).

### IFN-γ producers with no signs of cardiac disease exhibited low levels of circulating sCD127

**Fig 7:**
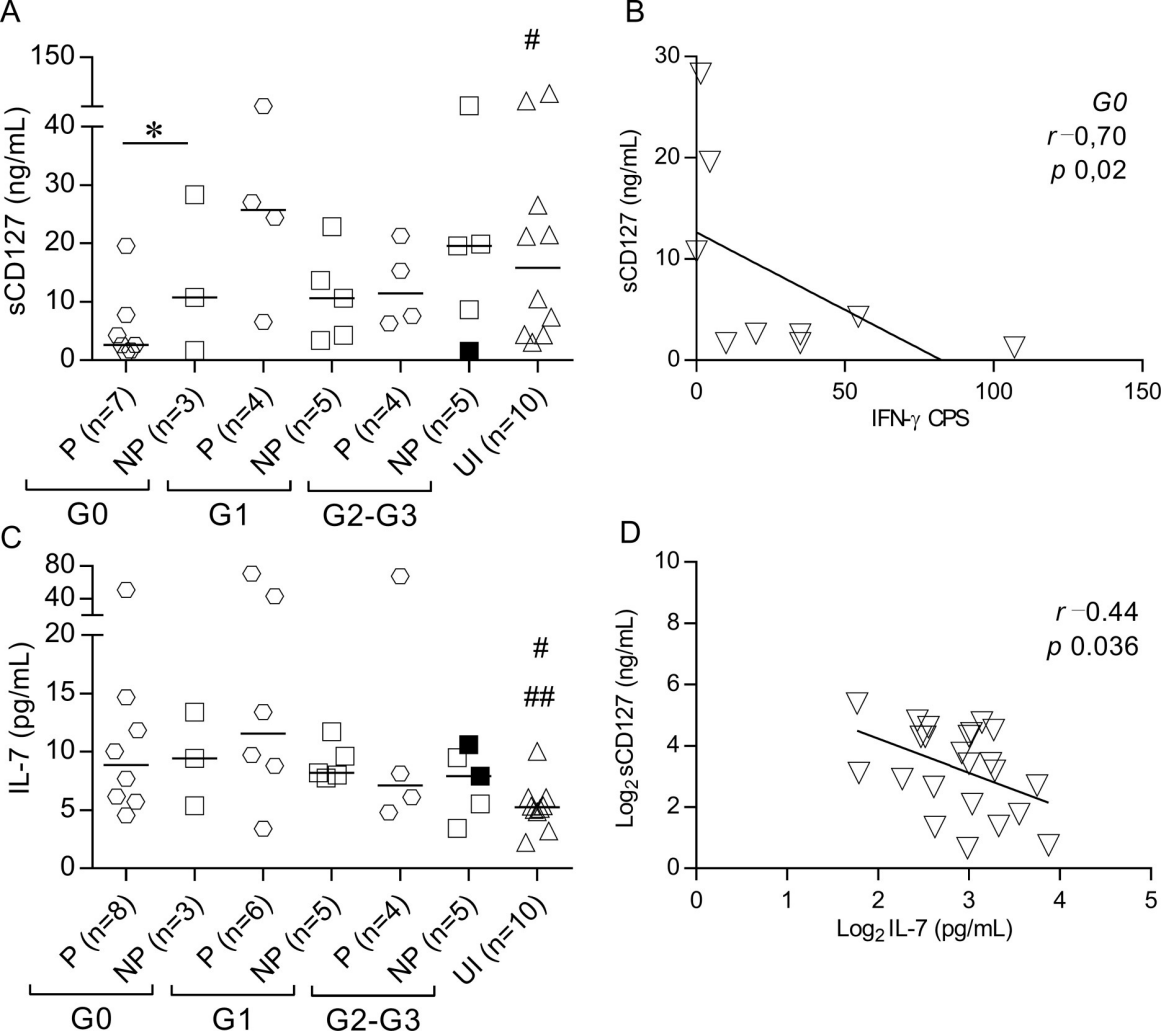
Decreased sCD127 serum levels in IFN-γ producers with no signs of cardiac dysfunction. Serum concentrations of sCD127 (A) and IL-7 (C) were measured using ELISA, and IFN-γ-producing cells were measured using ELISPOT in subjects at different clinical stages of chronic Chagas disease. Spearman’s correlation analysis between sCD127 and the *T*. *cruzi*-specific IFN-γ production in *T*. *cruzi*-infected subjects with no signs of cardiac dysfunction (B). Pearson’s correlation analysis between sCD127 and IL-7 in chronic Chagas disease patients (D). Horizontal lines indicate median values. Black symbols indicate subjects treated with benznidazole. Based on the ELISPOT assay, responses of *T*. *cruzi-*infected subjects were used to determine the IFN-γ producers (P) or IFN-γ nonproducers (NP), as described in Materials and Methods. Comparisons between P and NP for each clinical group and uninfected subjects were performed using ANOVA followed by Dunn’s multiple comparisons test. * p ≤ 0.05. (A) # p ≤ 0.05 compared with P G0; (C) # p ≤ 0.05 compared with P G0, NP G0 and P G1; ## p ≤ 0.01 compared with NP G1.

We investigated whether increased serum levels of IL-7 observed in patients with chronic Chagas disease [24] were associated with altered levels of the soluble form of the IL-7R (sCD127) and IFN-γ production. A lower sCD127 concentration was found in IFN-γ producers without signs of cardiac dysfunction than IFN-γ nonproducers and uninfected controls (Fig 7A) with a strong inverse correlation between sCD127 levels and the number of IFN-γ-producing cells (Fig 7B). In contrast, no differences were found in IL-7 levels between IFN-γ nonproducers and IFN-γ producers, and a weak inverse correlation was found between IL-7 and sCD127 serum levels (Fig 7C and 7D).

### Serum cytokines involved in regulation of the IL-7/IL7R pathway are altered in chronic Chagas disease

**Fig 8:**
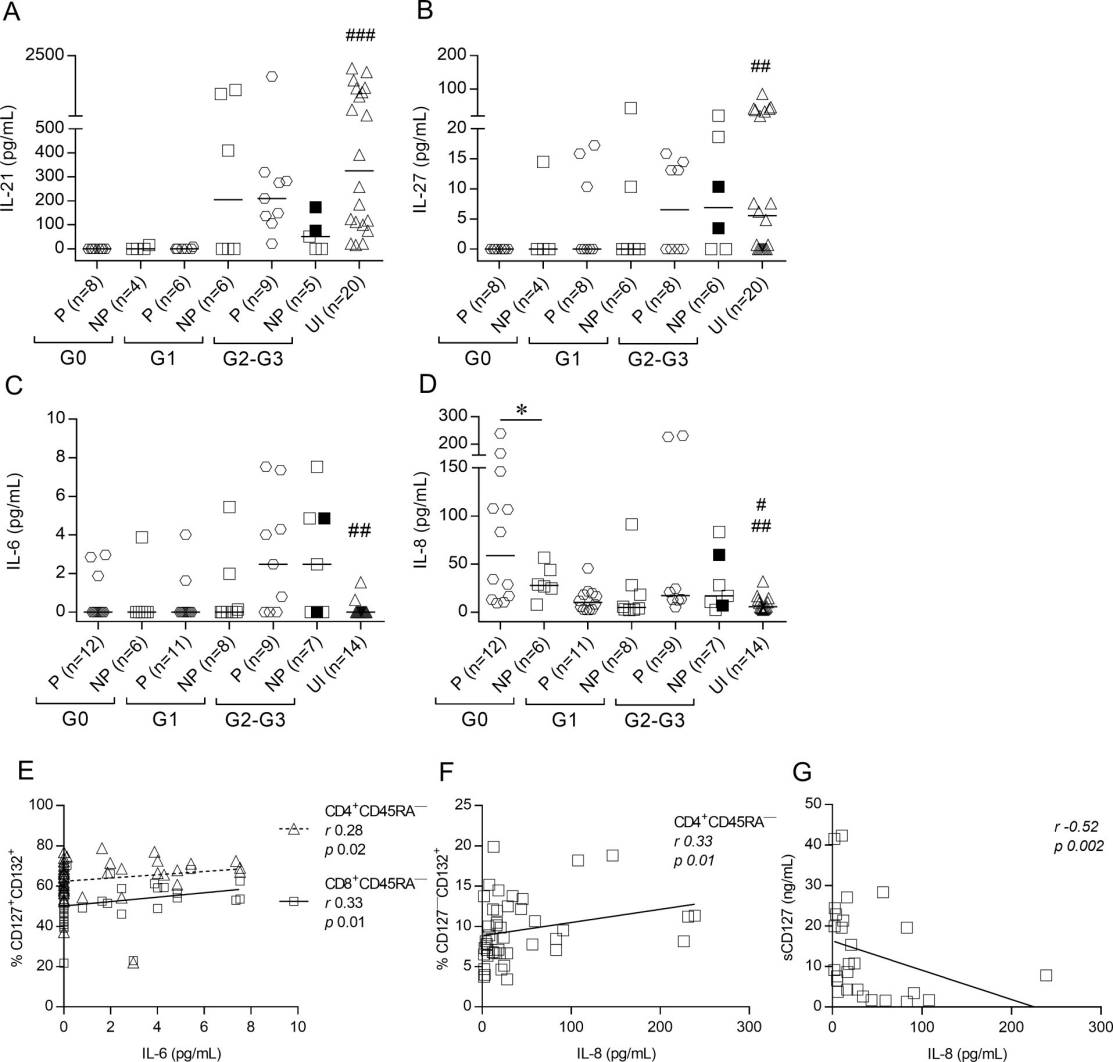
Serum cytokine levels are altered in subjects chronically infected with *T*. *cruzi*. IL-21, IL-27 and sCD127 serum levels were measured using ELISA, and IL-6 and IL-8 levels were measured using CBA. Each point represents the serum levels of IL-21 (A), IL-27 (B), IL-6 (C), and IL-8 (D). Values under the limit of detection are graphed as zero. Horizontal lines indicate median values. Black symbols indicate subjects treated with benznidazole. Based on the ELISPOT assay, responses of *T*. *cruzi-*infected subjects were used to determine IFN-γ producers (P) and IFN-γ nonproducers (NP), as described in Materials and Methods. Comparisons between P and NP for each clinical group and uninfected subjects were performed using ANOVA followed by Dunn’s multiple comparisons test. * p ≤ 0.05. (A) ### p ≤ 0.001 compared with P G0, NP G0 and P G1; (B) ## p ≤ 0.01 compared with P G0; (C) ## p ≤ 0.01 compared with P G2-G3 and NP G2-G3; (D) # p ≤ 0.05 compared with NP G0, ## p ≤ 0.01 compared with P G0. Correlation analysis between IL-6 levels and the frequency of CD127^+^CD132^+^ memory CD4^+^ and CD8^+^ T cells (E). IL-8 levels and the frequency of CD127^—^CD132^+^ memory CD4^+^ T cells (F). IL-8 levels and sCD127 (G) were assessed using Spearman correlation analysis.

Increased basal levels of T cells expressing phosphorylated STAT5 in IFN-γ nonproducers led us to hypothesize that other cytokines signaling through STAT5, including IL-9, IL-21 and IL-27, and inflammatory cytokines may be altered in these patients. IL-21, IL-27 and IL-6 levels were not significantly different between IFN-γ producers and IFN-γ nonproducers but varied according to disease severity (Fig 8A–8C, S5 Fig). Patients with a lower degree of cardiac dysfunction exhibited decreased levels of circulating IL-21 and IL-27 compared with patients with more severe disease and uninfected subjects (S5A and S5B Fig). Patients with severe cardiomyopathy exhibited increased IL-6 levels compared with patients with less severe forms of the disease and uninfected subjects (S5C Fig). IL-8 levels in patients with no signs of cardiac disease (i.e., the G0 group) were higher in IFN-γ producers than IFN-γ nonproducers and both groups were higher than uninfected subjects (Fig 8D). IL-6 levels positively correlated with the frequencies of CD4^+^ and CD8^+^ memory T cells with unmodulated CD127 (Fig 8E). In contrast, IL-8 levels positively correlated with the frequencies of CD4^+^ T cells with downregulated CD127 (Fig 8F) and inversely associated with sCD127 levels (Fig 8G). Serum concentrations of IL-1β, IL-9, IL-10, IL-12, and TNF-α were undetectable in most of the evaluated patients (S1 Table).

### Univariate and multivariate analysis of factors associated with *T*. *cruzi*-specific T-cell responses

Univariate and multivariate analyses were performed to identify independent parameters linked to the ability of T cells to produce IFN-γ in response to *T*. *cruzi* antigens regardless of the clinical status of the disease. Among T cells, a lower frequency of CD45RA^—^CD127^+^CD132^+^ cells, lower basal STAT5 phosphorylation and CD25 expression along with a higher frequency of CD45RA^—^CD127^—^CD132^+^ cells, higher frequency of phosphorylated STAT5 and CD25 expression after rhIL-7 stimulation, and higher basal Bcl-2 expression increased the likelihood of IFN-γ secretion in response to *T*. *cruzi* antigens (Table 2). In multivariate logistic regression analysis, the lower frequency of CD45RA^—^CD127^+^CD132^+^ cells, lower basal STAT5 phosphorylation and CD25 expression, along with higher basal Bcl-2 expression in T cells, were significant independent correlates of IFN-γ production in patients chronically infected with *T*. *cruzi* (Table 3). Notably, these associations were not uniform between CD4^+^ and CD8^+^ T cells (Tables 2 and 3).

**Table 2:** Univariate analysis of factors associated with effective T-cell responses in adults with chronic chagas disease.

Variable (Unit) ^A^	Univariate analysis		
	*T*. *cruzi*-specific IFN-γ production ^B^		p value ^C^
	IFN-γ producers	IFN-γ	
		nonproducers	
	(n = 32)	(n = 20)	
Age (years)	48 (42–57)	51 (43–57)	0.58
Sex (No. female/No. male)	14/18	7/13	0.13
CD4^+^ T cells			
CD45RA^—^CD127^+^CD132^+^ (%)	59 (54.4–66.5)	69 (66.1–72)	*<0*.*00001*
CD45RA^—^CD127^—^CD132^+^ (%)	37.7 (30.3–41.8)	26.9 (24.9–31.7)	*0*.*00001*
CD45RA^+^CD127^—^CD132^+^ (%)	36.9 (30–43.7)	35.8 (29.4–42.3)	0.33
CD45RA^+^CD127^+^CD132^—^(%)	0.9 (0.4–1.9)	0.9 (0.2–3.1)	0.93
Basal pSTAT5^+^ (%)	1.5 (0.9–2.54)	5.05 (1.6–9.2)	*0*.*00001*
Δ pSTAT5^+^ (%)	34.8 (14.8–64.9)	12.2 (0.003–36.9)	*0*.*01*
Basal CD25^+^ (%)	6.1 (4.4–7.8)	7.7 (3.9–11.05)	0.48
Δ CD25^+^ (%)	32.02 (22.7–34.6)	22.9 (5.9–33.9)	*0*.*043*
Basal Bcl-2^+^ (MFI)	108.3 (86.02–165)	71.3 (47.6–111)	*0*.*0084*
Δ Bcl-2^+^ (MFI)	20.3 (7.7–40)	17.2 (0.5–36)	0.60
CD8^+^ T cells			
CD45RA^—^CD127^+^CD132^+^ (%)	46.01 (41–51.4)	60.2 (56.05–66.2)	*<0*.*00001*
CD45RA^—^CD127^—^CD132^+^ (%)	50.2 (45.3–55.1)	35.3 (30.9–40)	*<0*.*00001*
CD45RA^+^CD127^—^CD132^+^ (%)	47.7 (39.3–56.2)	43.9 (34.3–51.2)	0.79
CD45RA^+^CD127^+^CD132^—^(%)	1.5 (0.8–2.1)	1.9 (0.85–2.9)	0.22
Basal pSTAT5^+^ (%)	3.3 (1.45–3.9)	5.85 (2.9–6.9)	*0*.*00001*
Δ pSTAT5^+^ (%)	20.9 (8.8–44.1)	5.2 (0.8–18)	*0*.*004*
Basal CD25^+^ (%)	2.70 (1.6–3.6)	7.4 (4.05–12.3)	*<0*.*00001*
Δ CD25^+^ (%)	20.6 (12.3–26.1)	13.5 (6.6–22.6)	*0*.*036*
Basal Bcl-2^+^ (MFI)	97.4 (86.4–137.25)	87.6 (47–128)	0.19
Δ Bcl-2^+^ (MFI)	22.7 (12.47–29.75)	16.8 (1–39)	0.84
Soluble Factors in Sera			
sCD127 (ng/mL)	7.7 (2.6–21.3)	10.6 (3.6–21.40)	0.70
IL-6 (pg/mL)	0 (0–2.1)	0 (0–4.8)	0.17
IL-7 (pg/mL)	9.3 (6.05–21.8)	8.1 (7.2–9.9)	0.38
IL-8 (pg/mL)	18.3 (10.4–106.7)	21.4 (6.2–46.9)	0.66
IL-21 (pg/mL)	0 (0–172)	33.3 (0–232.2)	0.34
IL-27 (pg/mL)	0 (0–13.1)	0 (0–15.00)	0.43

^A^ Data for continuous variables are shown as medians (interquartile range).

^B^ A positive response to *T*. *cruzi* antigens comprises i) a minimum of 10 spots/4 × 10^5^ PBMCs present per *T*. *cruzi*-stimulated well in response to *T*. *cruzi* antigens, and ii) the number of spots in stimulated wells must be at least twice the number of spots in wells with medium alone in an ELISPOT assay. Both conditions must be met to consider a subject as an IFN-γ producer.

^C^ Italic values indicate p values < 0.05.

**Table 3:** Independent correlates of functional T-cell responses.

Variable (Unit) ^A^	Multivariate analysis ^B^		
	Odds ratio	95% CI	p value
CD4^+^CD45RA^—^CD127^+^CD132^+^ (%)	0.65	0.49–0.87	*0*.*003*
Basal CD4^+^pSTAT5^+^ (%)	0.28	0.12–0.65	*0*.*003*
Basal CD4^+^Bcl-2^+^ (MFI)	1.04	1.01–1.08	*0*.*013*
CD8^+^CD45RA^—^CD127^+^CD132^+^ (%)	0.71	0.55–0.9	*0*.*005*
Basal CD8^+^CD25^+^ (%)	0.44	0.23–0.87	*0*.*017*

^A^ Data for continuous variables are shown as medians (interquartile range).

^B^ Variables were grouped into six different models. In each model, there was no correlation between variables according to Spearman’s correlation analysis.

Correlation analyzes were performed to evaluate differences in the expression of the parameters evaluated according to the magnitude of *T*. *cruzi*-specific responses in the group of IFN-γ producers. The number of IFN-γ CPS in patients in the G0 and G1 groups positively correlated with the percentages of memory CD8^+^ T cells with unmodulated CD127 (i.e., CD45RA^—^CD127^+^CD132^+^) and inversely correlated with the percentages of memory CD8^+^ T cells with downregulated CD127 (i.e., CD45RA^—^CD127^—^CD132^+^) (Table 4). The number of IFN-γ CPS in G0 patients also positively associated with the basal expression of Bcl-2 in T cells and inversely associated with IL-7 serum concentration (Table 4). An inversely correlation was also observed between IFN-γ CPS and the basal percentages of CD8^+^pSTAT5^+^ in G1 patients (Table 4). IFN-γ CPS in patients in the G2 and G3 groups positively correlated with the percentages of TTE CD4^+^ and CD8^+^ T cells and inversely correlated with CD4^+^RTE (Table 4).

**Table 4:** Correlation analyses between the number of IFN-γ producing cells among IFN-γ producers and the parameters associated with IL-7/IL-7R axis.

IFN-γ CPS among IFN-γ Producers *vs*.^A^	Clinical Group	Spearman r	p value
Basal MFI CD4^+^Bcl-2^+^	G0	0.83	*0*.*007*
% CD4^+^CD45RA^+^CD127^—^CD132^+^	G2-G3	0.56	*0*.*038*
% CD4^+^CD45RA^+^CD127^+^CD132^—^	G2-G3	-0.53	*0*.*048*
% CD8^+^CD45RA^—^CD127^+^CD132^+^	G0	0.58	*0*.*038*
	G1	0.80	*0*.*006*
% CD8^+^CD45RA^—^CD127^—^CD132^+^	G0	-0.67	*0*.*019*
	G1	-0.70	*0*.*021*
% CD8^+^CD45RA^+^CD127^—^CD132^+^	G2-G3	0.61	*0*.*042*
Basal % CD8^+^pSTAT5^+^	G1	-0.68	*0*.*025*
IL-7 serum levels	G0	-0.85	*0*.*011*

^A^ Correlations were evaluated by Spearman’s test.

### Low levels of IFN-γ-producing cells cannot be reverted with in vitro treatment with IL-7 or IL-27

**Fig 9:**
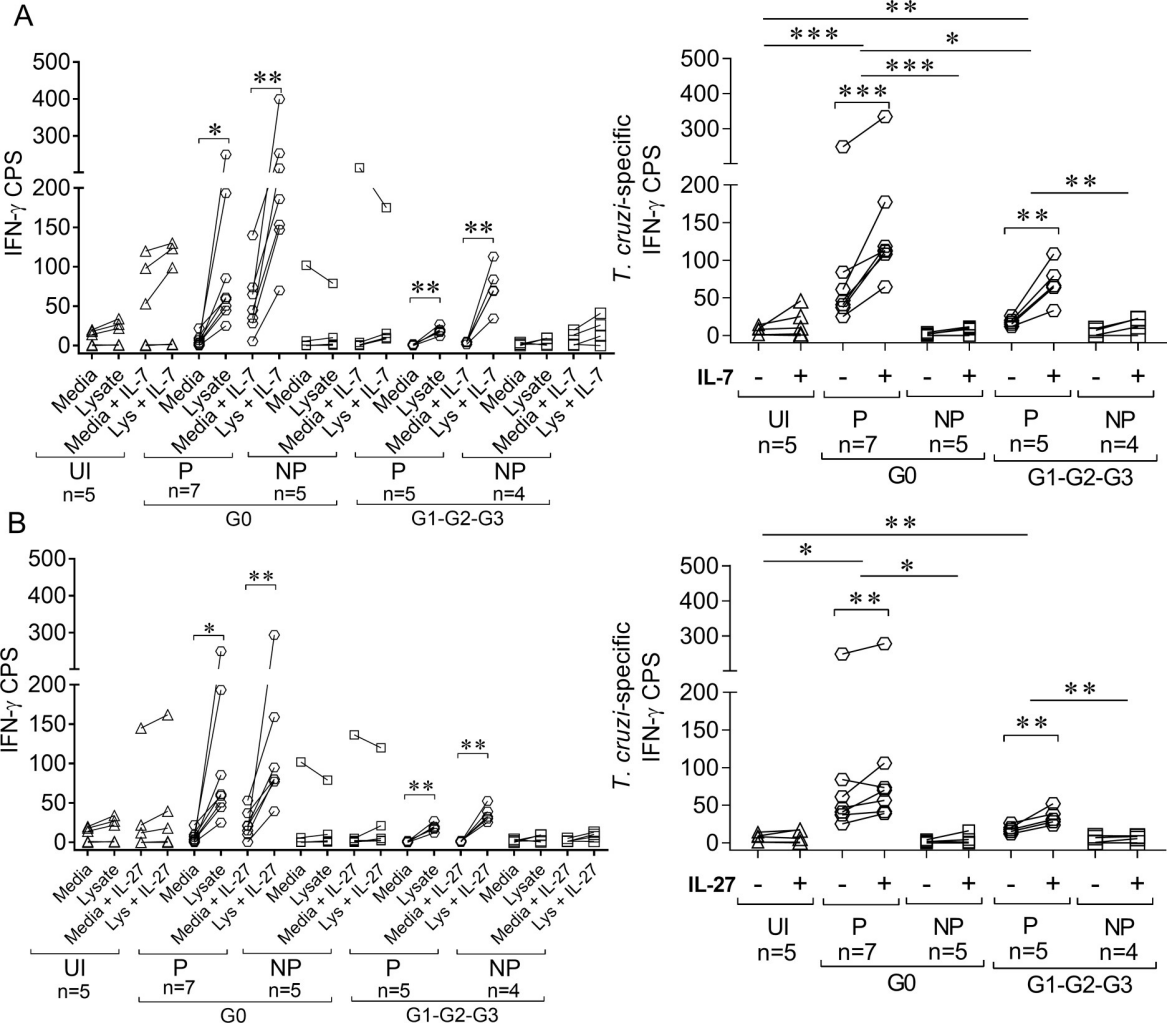
Low IFN-γ production was not reversed after short-term IL-7 or IL-27 treatment. For IFN-γ ELISPOT assays, PBMCs were seeded at 4 × 10^5^ cells/well and stimulated with 10 μg/mL *T*. *cruzi* lysate or media alone in the presence or absence of 50 ng/mL IL-7 (A, left panel) or 50 ng/mL IL-27 (B, left panel) for 16–20 h. The number of *T*. *cruzi*-specific IFN-γ–secreting T cells in the presence of IL-7 (A, right panel) or IL-27 (B, right panel) was calculated by subtracting the value of wells containing media alone from the *T*. *cruzi* lysate-stimulated spot count. Paired *t*-test was used to compare the number of IFN-γ cells producing spots (CPS) between the unstimulated and *T*. *cruzi*-stimulated wells (A and B, left panel) or the number of *T*. *cruzi*-specific IFN-γ CPS in presence (+) or absence (-) of cytokines and are indicated by brackets. The differences in the number of IFN-γ CPS in IL-7- and IL-27-stimulated and unstimulated samples were compared between clinical groups using the Mann-Whitney U test and indicated by lines. * p ≤ 0.05, ** p ≤ 0.01, *** p ≤ 0.001, UI: uninfected, P: IFN-γ producers, NP: IFN-γ nonproducers.

**Fig 10:**
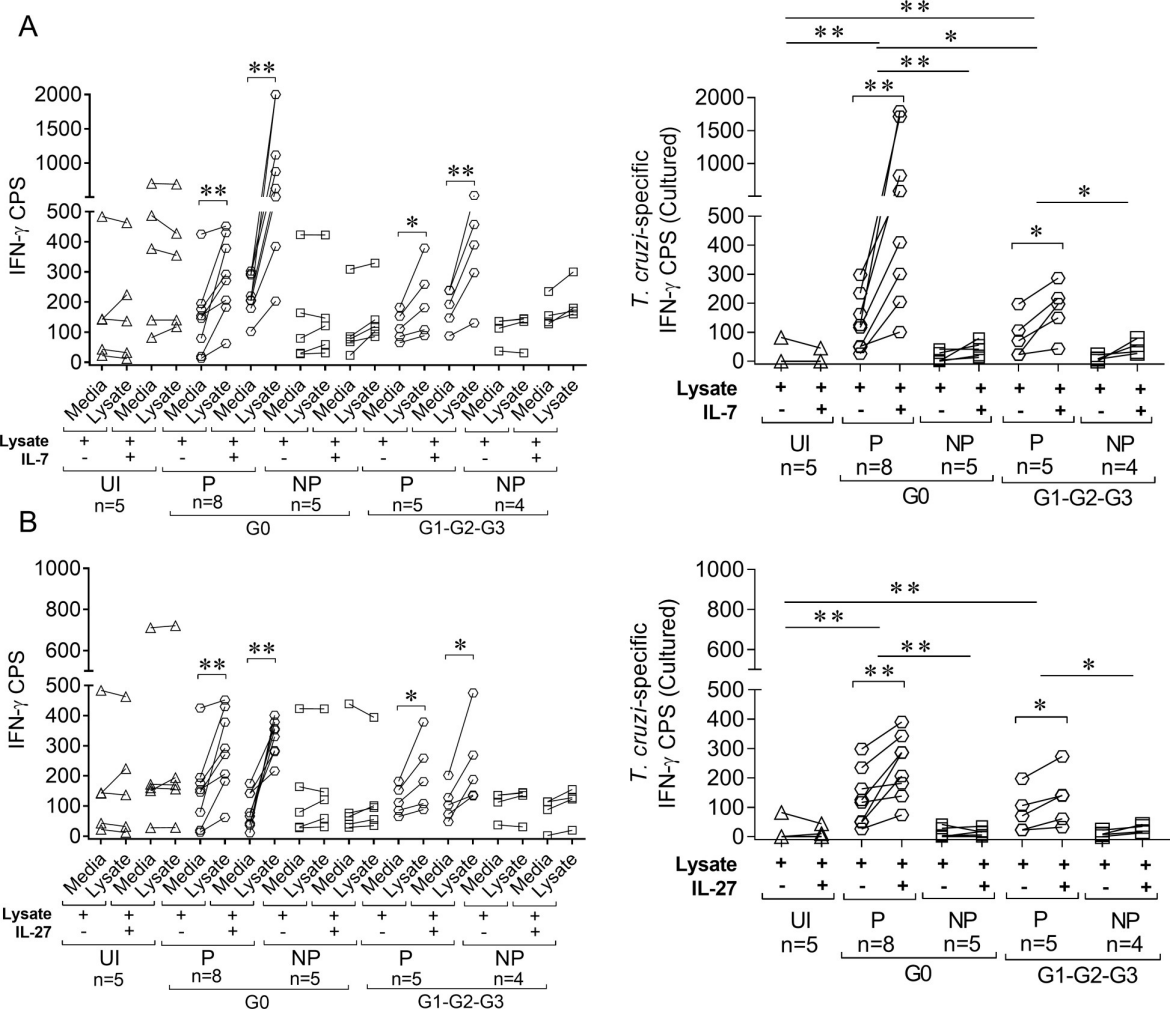
Low IFN-γ production was not reversed after 10 days of cell culture with IL-7 or IL-27. PBMCs were cultured for 10 days with *T*. *cruzi* lysate in the presence or absence of IL-7 (A) or IL-27 (B). For IFN-γ ELISPOT assays, cultured PBMCs were seeded at 2 × 10^5^ cells/well with the addition of 1 × 10^5^ autologous PBMCs as antigen-presenting cells and restimulated with 10 μg/mL *T*. *cruzi* lysate or media alone (A and B, left panel) for 16–20 h. The number of *T*. *cruzi*-specific IFN-γ–secreting T cells was calculated by subtracting the value of wells containing media alone from the *T*. *cruzi* lysate-stimulated spot count (A and B, right panel). Paired *t*-test was used to compare the number of IFN-γ cells producing spots (CPS) between unstimulated and *T*. *cruzi*-stimulated wells (A and B, left panel) and the number of *T*. *cruzi*-specific IFN-γ CPS after *in vitro* culture with T. cruzi lysate for 10 days in the presence (+) or absence (-) of IL-7 or IL-27 and are indicated by brackets. The differences in the numbers of IFN-γ CPS in IL-7 or IL-27 stimulated and unstimulated samples were compared between clinical groups using Mann-Whitney U test and indicated by lines. * p ≤ 0.05, ** p ≤ 0.01, *** p ≤ 0.001, UI: uninfected, P: IFN-γ producers, NP: IFN-γ nonproducers.

We examined whether *in vitro* treatment of PBMCs with IL-7 or IL-27 enhanced *T*. *cruzi*-specific T cell responses in chronic Chagas disease patients. Addition of IL-7 or IL-27 in short-term cultures with *T*. *cruzi* antigens increased the number of IFN-γ-producing T cells in response to *T*. *cruzi* in IFN-γ producers but not IFN-γ nonproducers in patients with no signs of cardiac disease and patients with some degree of cardiac dysfunction. This increase was specific because no changes in the frequencies of *T*. *cruzi*-responsive IFN-γ-producing cells were observed in uninfected subjects (Fig 9A and 9B). A significant expansion of IFN-γ-producing cells were obtained after a 10-day ex vivo culture with *T*. *cruzi* antigens following the addition of IL-7 or IL-27 in IFN-γ producers, and IFN-γ-producing cells remained unchanged in IFN-γ nonproducers (Fig 10A and 10B). Notably, the fold increase in IFN-γ producers with cardiac dysfunction was significantly lower than IFN-γ producers without cardiac disease (Fig 9A and 9B, right panels; Fig 10A and 10B, right panels).

## Discussion

The IL-7/IL-7R signaling pathway is necessary for memory T-cell formation, homeostasis, and self-renewal after resolution of an acute infection. In contrast, T cells fail to develop into self-renewing, antigen-independent memory T cells during chronic infections, which may be driven by the reduced expression of IL-7R on T cells or a failure to respond efficiently to this cytokine, with a subsequent loss of pathogen-specific T cells [31–36]. The present study demonstrated that the ability of T cells to secrete IFN-γ in response to *T*. *cruzi* was associated with a functional IL-7/IL-7R signaling pathway in memory T cells, high basal expression of Bcl-2, low basal levels of activated T cells, and fewer inhibitory mechanisms on this axis, regardless the clinical stage of the disease. Grouping of patients according to the clinical stage revealed striking differences in the IL-7/IL-7R pathway in IFN-γ producers and IFN-γ nonproducers. The downregulation of IL-7Rα chain expression in memory T cells decayed with increasing disease severity in IFN-γ producers. Notably, the number of IFN-γ producing cells in IFN-γ producers with less severe forms of the disease was inversely associated with the frequency of memory T cells with downregulated CD127, serum levels of IL-7 and the basal frequencies of pSTAT5^+^ T cells and positively associated with the basal expression of the anti-apoptotic molecule Bcl-2. The number of IFN-γ producing cells in IFN-γ producers with severe cardiomyopathy was positively associated with TTE levels and inversely with the frequencies of RTE cells. These findings support the hypothesis that *T*. *cruzi*-specific T-cell responses are maintained at least partially via recruitment from RTE cells, which is consistent with the decreased frequencies of naïve T cells [11, 37] and the low degree of differentiation in IFN-γ-producing cells [13] from individuals chronically infected with *T*. *cruzi* subjects.

The increased recruitment of RTE in patients with more severe forms of the disease may be a compensatory mechanism to maintain parasite-specific T cells. Increased IL-7 levels may be responsible for the increased basal levels of pSTAT5^+^ and CD25^+^ T cells as part of these compensatory mechanisms to counteract the disruption in the IL-7/IL-7R axis. Fonseca et al. demonstrated high mRNA expression of IL-7 in heart tissues of patients with Chagas disease cardiomyopathy [38]. *T*. *cruzi*-infected subjects lacking parasite-specific T-cell responses and showing no signs of cardiac dysfunction still exhibited a functional signature of the IL-7/IL-7R pathway in T cells with STAT5 phosphorylation and CD25 expression in response to IL-7. In contrast, IFN-γ nonproducers with severe cardiomyopathy exhibited an impaired capacity to respond to IL-7. We confirmed that IFN- γ-producing cells in response to *T*. *cruzi* exhibited downregulated CD127 expression.

IL-7R plays a role in IL-7 signaling during homeostasis, and it is rapidly internalized and recycled to the cell surface without changing T cell phenotype [39]. IL-7R regulation is also controlled at the transcriptional level, and IL-7 and other cytokines suppress IL-7R mRNA expression [40]. The role of the soluble form of IL-7R (sCD127), which is generated by cleavage or alternative splicing [41], is controversial. Some reports demonstrated the sCD127 inhibits IL-7 activity [19, 21, 42], and other studies demonstrated that IL-7 in complex with sCD127 delivered a more potent signal to cell-bound IL-7Rs or constituted a reservoir of IL-7 [43–44]. Increased serum levels of IL-7 in patients with less severe forms of Chagas disease may be induced by the need to maintain T cells during chronic infection because receptor-mediated uptake largely regulates IL-7 levels [45]. Increased IL-7 levels may inhibit IL-7R expression and the release of sCD127, which was supported by the negative correlation between IL-7 and sCD127. This scenario, sustained over time, may lead to desensitization of the pathway, which would result in losses of pathway regulation and T-cell responses, as part of the mechanism of immune exhaustion. This hypothesis was particularly demonstrable in IFN-γ producers with severe cardiomyopathy, who exhibit normal values of IL-7 and sCD127 but decreased responses to IL-7 by the inverse association between IFN-γ producing cells and circulating sCD127.

Our previous observations and other studies support the process of immune exhaustion and demonstrated that patients with no signs of cardiac dysfunction exhibited higher frequencies of circulating IFN-γ-producing cells compared with patients with severe cardiomyopathy [10, 14; 46]. Several mechanisms underlying immune exhaustion in chronic Chagas disease were described, including the lack proliferative capacity and downregulation of CD28 and CD3ζ [47], increased nitric oxide production concomitant with increased tyrosine nitration [48] and increased expression of inhibitory receptors in T cells [13–14; 49–50]. The present study demonstrated that *T*.*cruzi*-induced IFN-γ-producing cells expressed high levels of PD-1, which is a primary inhibitory receptor associated with immune exhaustion [51–53], in addition to CTLA-4 and LIR-1 [13]. Our data also demonstrated a positive correlation between IL-6 levels and T cells with unmodulated CD127, which suggests that this inflammatory cytokine blocks T-cell responses to IL-7, as found in HIV infection [22–23]. Notably, inflammatory cytokines and IL-7 induce the expression of exhaustion and senescence markers, PD-1 and CD57, on T cells [23]. Several studies in subjects chronically infected with *T*. *cruzi* revealed that increased levels of IL-6 were associated with cardiac dysfunction [54–57], which supports the hypothesis that sustained inflammation in the chronic phase of infection may also alter the homeostatic mechanisms of T-cell maintenance.

IL-8 is a monocyte-derived cytokine that is upregulated by IL-7 [58], and it was increased in IFN-γ producers with no signs of cardiac dysfunction and inversely associated with sCD127. These results suggest that IL-7 also plays a critical role in the regulation of macrophage cytokine expression. However, other pathways besides IL-7/IL-7R may be involved in the maintenance of T-cell responses. We observed that subjects with a functional IL-7 axis lacked *T*. *cruzi*-responsive T cells. The IL-21 and IL-27 levels, which also signal via STAT5 and induce T-cell proliferation and effector function [59–61], were also altered in individuals chronically infected with *T*. *cruzi*. IL-21 and IL-27 appeared to be consumed in IFN-γ-producers with less severe forms of the disease, which may be another mechanism for the maintenance of T-cell responses.

Exogenous addition of IL-7 or IL-27 did not rescue *T*. *cruzi*-responsive IFN-γ-producing T cells in patients with undetectable IFN-γ-producing cells, which suggests that *T*. *cruzi*-specific T cells are present but the JAK/STAT pathway is dysfunctional or that *T*. *cruzi*-specific T cells were already depleted from the circulation. The higher fold-increase in *T*. *cruzi*-specific T cells in IFN-γ responders with no signs of cardiac dysfunction compared with patients with severe disease further supports the hypothesis that impairment in T-cell function is a gradual process. Therefore, our findings provide new insights into the regulation of T cell-mediated immunity against *T*. *cruzi*-infection and may aid the design of a vaccine against *T*. *cruzi*. However, it is not possible to ascertain whether the impairment of *T*. *cruzi*-specific T-cell responses is a cause or a consequence of disease progression. Another limitation of the present study is that the T-cell responses focused solely on responses elicited by a *T*. *cruzi* lysate, for which the bulk of the response is CD4^+^. Unfortunately, CD8^+^ T-cell responses elicited by HLA-restricted *T*. *cruzi*-derived epitopes are of very low frequencies [12].

Impaired T-cell responses in chronic Chagas disease are specific for *T*. *cruzi*, but these alterations in the IL-7/IL-7R pathway are another example of how this chronic infection affects the general status of the host immune system. Taken together, the present study demonstrated that defective signaling and regulatory mechanisms in the IL-7/IL-7R axis during the chronic phase of Chagas disease may affect the maintenance of parasite-specific IFN-γ-producing cells.

## Supporting information

S1 Fig Cell-surface expression of IL-7R components in CD4^+^ and CD8^+^ T cells in IFN-γ producers and IFN-γ nonproducers in response to *T. cruzi* antigens. PBMCs were stained for FV510, CD4/CD8, CD45RA, CD127, and CD132 and analyzed using flow cytometry. Lymphocytes were gated by side scatter versus forward scatter channels and subsequently analyzed by CD4/CD8 vs. CD45RA (A). Data were analyzed according to minus one controls for CD127 (left panel) and CD132 (right panel) (B). The pattern of CD127 and CD32 expression on CD45RA^—^(C, D) and CD45RA^+^ (E, F) among CD4^+^ (left panel) and CD8^+^ (right panel) T cells was then analyzed. Representative dot plots of one IFN-γ producer (P), one IFN-γ non-producer (NP) and one uninfected control (UI), as defined in Materials and Methods, are shown.
(TIF)

S2 Fig Frequency of memory CD4^+^ and CD8^+^ T cells with downregulated CD127 diminish with increased disease severity. PBMCs were stained with FV510, CD45RA, CD8, CD4, CD127, and CD132 monoclonal antibodies and analyzed using flow cytometry. *T*. *cruzi-*specific T-cell responses were determined using IFN-γ ELISPOT after stimulation of PBMCs with a *T*. *cruzi* lysate. Each symbol represents the proportion of CD127^+/—^CD132^+^ cells among total CD4^+^CD45RA^—^(A and B) or CD8^+^CD45RA^—^(C and D) T-cell populations. Median values are indicated as horizontal lines. The responses of *T*. *cruzi*-infected subjects were used to determine the IFN-γ producers and IFN-γ nonproducers based on the ELISPOT assay, as described in Materials and Methods. Oblique lines indicate a significant tendency between medians by testing for a linear trend. A, p = 0.03 slope: 4.85; B, p = 0.025 slope: -4.84; C, p = 0.023 slope: 3.87; D, p = 0.03 slope: -3.83.
(TIF)

S3 Fig Gate strategy for the analysis of IL-7R components and PD-1 expression among CD4^+^ T cells following stimulation with *T. cruzi* lysate. PBMCs were stimulated for 18–20 h with *T*. *cruzi* lysate (E), media alone (D) or SEB (F). Cells were stained with FV510, CD4, CD127, CD132 and PD-1 monoclonal antibodies followed by fixation and permeabilization for intracellular staining with an anti-IFN-γ monoclonal antibody. Representative dot plots of the gating strategy are shown. Lymphocytes were gated based on forward (FSC) and side scattering (SSC) (A). Single cells were selected based on FSC-W and FSC-A (B), and viable cells were gated by their negative staining for the viability marker FV510 (C). CD4^+^ T cells were analyzed for IFN-γ expression. CD127, CD132 and PD-1 expression was analyzed on IFN-γ-producing (E) and IFN-γ nonproducing (D) CD4^+^ T cells.
(TIF)

S4 Fig Interleukin-7-mediated signaling through STAT5 in IFN-γ producers and nonproducers in response to *T. cruzi* antigens. PBMCs were stimulated with 100 ng/mL IL-7 and evaluated forpSTAT5 induction in CD4^+^ and CD8^+^ T cells by flow cytometry. Lymphocytes were gated in side scatter versus forward scatter channels. Representative CD4^+^ and CD8^+^ histogram plots show PBMCs from an IFN-γ producer (P, A and C) and a non-producer (NP, B and D), as described in Materials and Methods. Slashed gray lines indicate the basal expression of pSTAT5, and black lines indicate the expression of pSTAT5 after IL-7 stimulation.
(TIF)

S5 Fig Altered serum IL-21, IL-27 and IL-6 levels in chronic Chagas disease patients. IL-21 and IL-27 were measured using ELISA, and IL-6 levels were measured using CBA. Each point represents the serum levels of IL-21 (A), IL-27 (B) and IL-6 (C) of individual subjects. Values under the limit of detection were graphed as zero. Horizontal lines indicate median values. Black symbols indicate subjects treated with benznidazole. Comparisons between clinical groups and uninfected subjects were performed using ANOVA followed by Dunn’s multiple comparison test. * p ≤ 0.05, ** p ≤ 0.01, *** p ≤ 0.001 compared with G2-G3. (A) ### p ≤ 0.001 compared with G0 and G1; (B) ## p ≤ 0.01 compared with G0; (C) ## p ≤ 0.01 compared with G2-G3.
(TIF)

S1 Table Cytokines serum levels in chronic Chagas disease patients. (PDF)
