# Correction: *Trypanosoma cruzi*-specific IFN-γ-producing cells in chronic Chagas disease associate with a functional IL-7/IL-7R axis

**DOI:** 10.1371/journal.pntd.0007168

**Published:** 2019-02-26

**Authors:** María A. Natale, Gonzalo Cesar, Maria G. Alvarez, Melisa D. Castro Eiro, Bruno Lococo, Graciela Bertocchi, María C. Albareda, Susana A. Laucella

The second author's name is spelled incorrectly. The correct name is: Gonzalo Cesar. The correct citation is: Natale MA, Cesar G, Alvarez MG, Castro Eiro MD, Lococo B, Bertocchi G, et al. (2018) *Trypanosoma cruzi*-specific IFN-γ-producing cells in chronic Chagas disease associate with a functional IL-7/IL-7R axis. PLoS Negl Trop Dis 12(12): e0006998. https://doi.org/10.1371/journal.pntd.0006998
