# Supplementary figures and images for: Trypanosoma cruzi-specific IFN-γ-producing cells in chronic Chagas disease associate with a functional IL-7/IL-7R axis
Source: PLoS Negl Trop Dis. 2018 Dec 5;12(12):e0006998. doi: 10.1371/journal.pntd.0006998 (PMC6281225; doi:10.1371/journal.pntd.0006998)

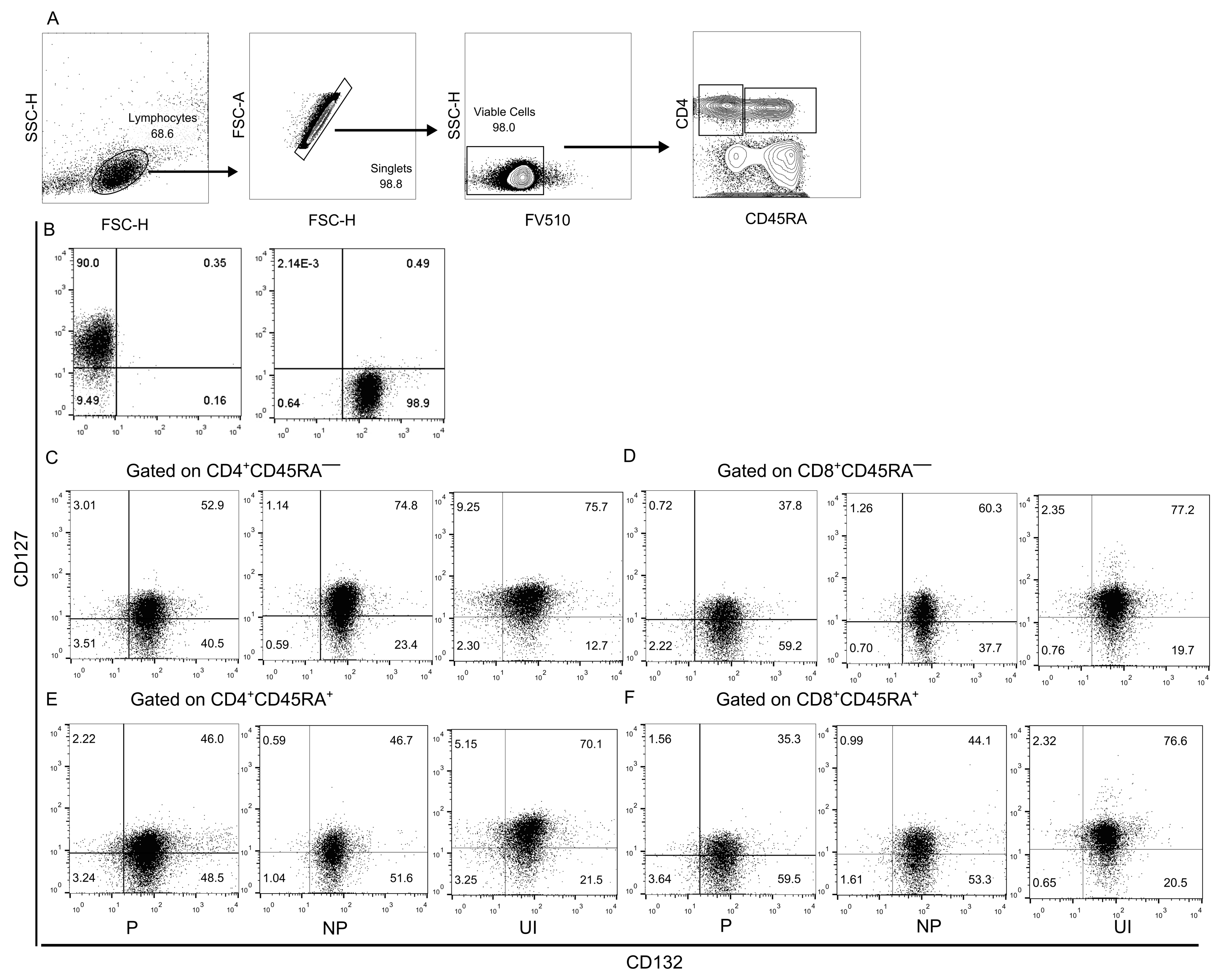

Supplement: S1 Fig — PBMCs were stained for FV510, CD4/CD8, CD45RA, CD127, and CD132 and analyzed using flow cytometry. Lymphocytes were gated by side scatter versus forward scatter channels and subsequently analyzed by CD4/CD8 vs. CD45RA (A). Data were analyzed according to minus one controls for CD127 (left panel) and CD132 (right panel) (B). The pattern of CD127 and CD32 expression on CD45RA—(C, D) and CD45RA+ (E, F) among CD4+ (left panel) and CD8+ (right panel) T cells was then analyzed. Representative dot plots of one IFN-γ producer (P), one IFN-γ non-producer (NP) and one uninfected control (UI), as defined in Materials and Methods, are shown. (TIF) [file pntd.0006998.s001.tif]

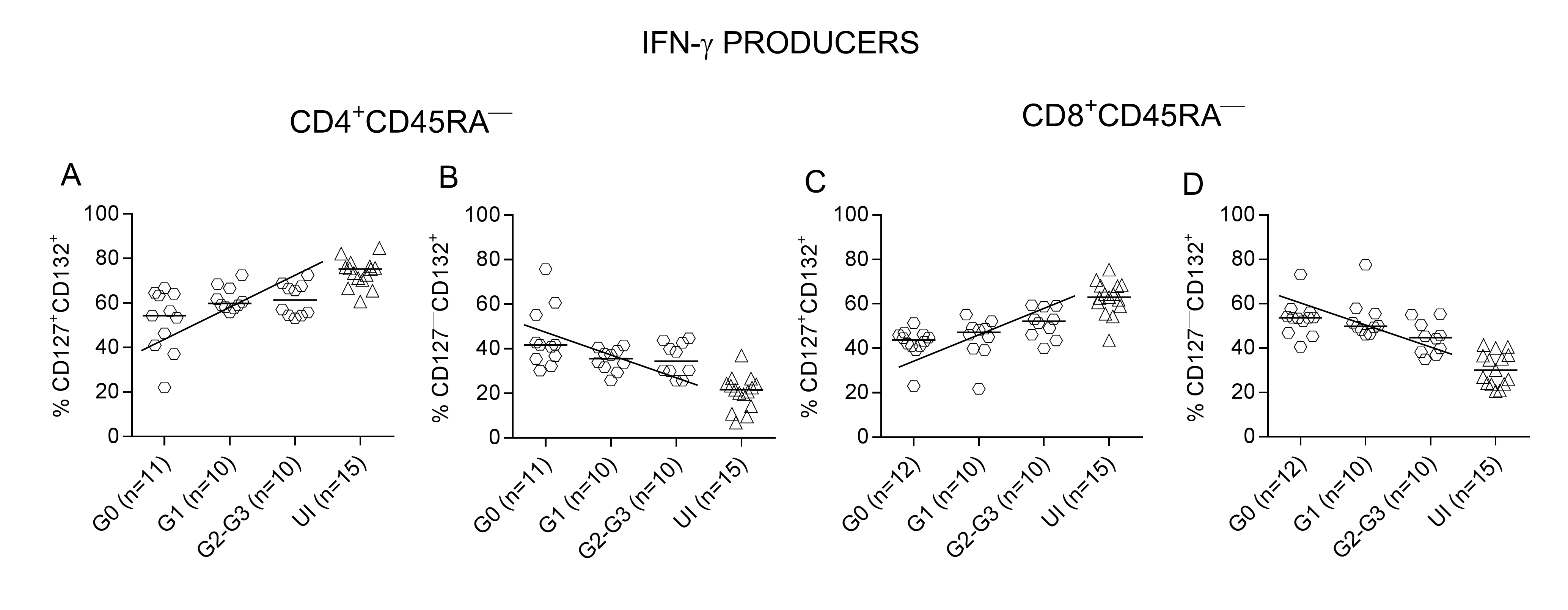

Supplement: S2 Fig — PBMCs were stained with FV510, CD45RA, CD8, CD4, CD127, and CD132 monoclonal antibodies and analyzed using flow cytometry. T. cruzi-specific T-cell responses were determined using IFN-γ ELISPOT after stimulation of PBMCs with a T. cruzi lysate. Each symbol represents the proportion of CD127+/—CD132+ cells among total CD4+CD45RA—(A and B) or CD8+CD45RA—(C and D) T-cell populations. Median values are indicated as horizontal lines. The responses of T. cruzi-infected subjects were used to determine the IFN-γ producers and IFN-γ nonproducers based on the ELISPOT assay, as described in Materials and Methods. Oblique lines indicate a significant tendency between medians by testing for a linear trend. A, p = 0.03 slope: 4.85; B, p = 0.025 slope: -4.84; C, p = 0.023 slope: 3.87; D, p = 0.03 slope: -3.83. (TIF) [file pntd.0006998.s002.tif]

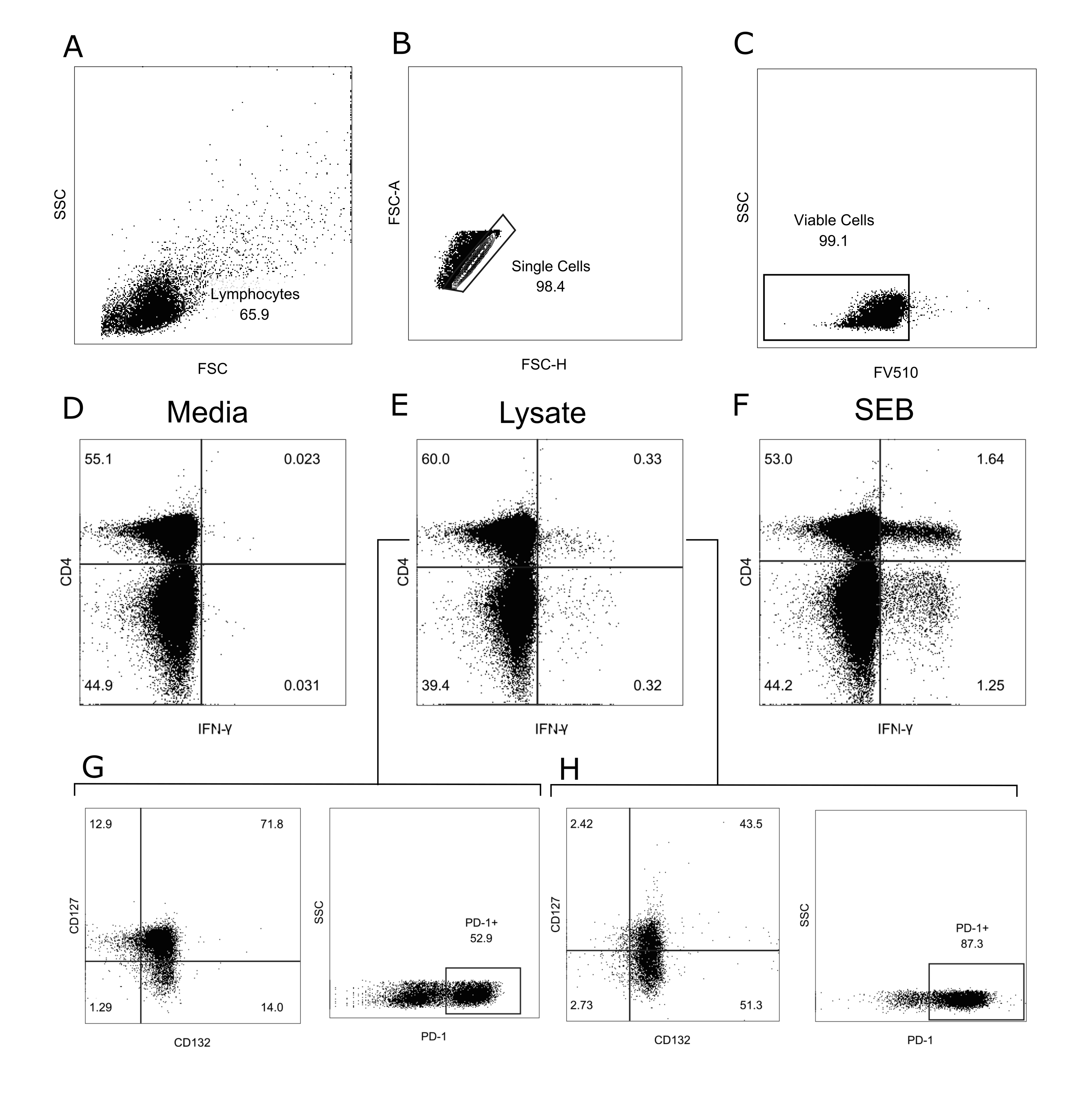

Supplement: S3 Fig — PBMCs were stimulated for 18–20 h with T. cruzi lysate (E), media alone (D) or SEB (F). Cells were stained with FV510, CD4, CD127, CD132 and PD-1 monoclonal antibodies followed by fixation and permeabilization for intracellular staining with an anti-IFN-γ monoclonal antibody. Representative dot plots of the gating strategy are shown. Lymphocytes were gated based on forward (FSC) and side scattering (SSC) (A). Single cells were selected based on FSC-W and FSC-A (B), and viable cells were gated by their negative staining for the viability marker FV510 (C). CD4+ T cells were analyzed for IFN-γ expression. CD127, CD132 and PD-1 expression was analyzed on IFN-γ-producing (E) and IFN-γ nonproducing (D) CD4+ T cells. (TIF) [file pntd.0006998.s003.tif]

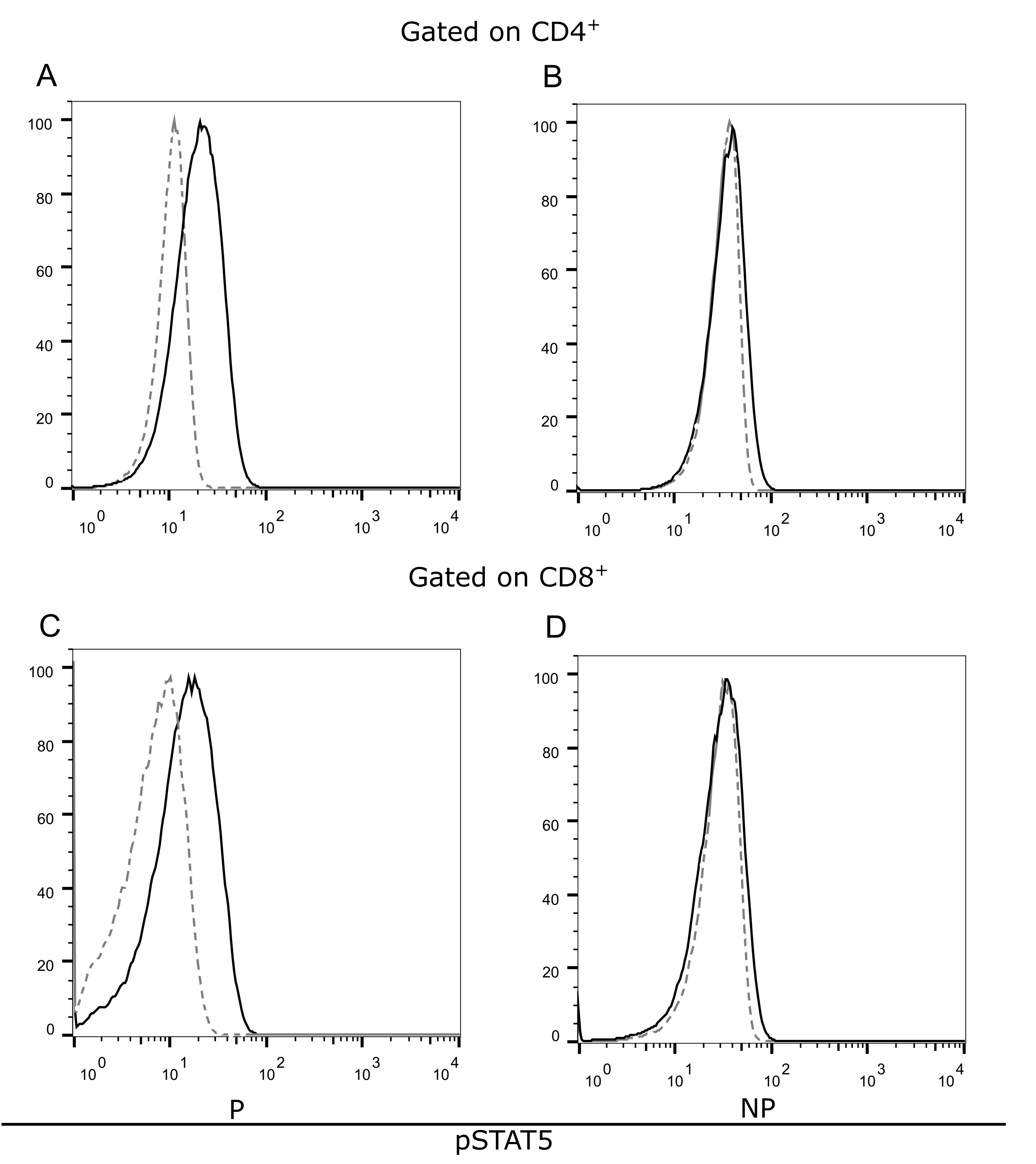

Supplement: S4 Fig — PBMCs were stimulated with 100 ng/mL IL-7 and evaluated forpSTAT5 induction in CD4+ and CD8+ T cells by flow cytometry. Lymphocytes were gated in side scatter versus forward scatter channels. Representative CD4+ and CD8+ histogram plots show PBMCs from an IFN-γ producer (P, A and C) and a non-producer (NP, B and D), as described in Materials and Methods. Slashed gray lines indicate the basal expression of pSTAT5, and black lines indicate the expression of pSTAT5 after IL-7 stimulation. (TIF) [file pntd.0006998.s004.tif]

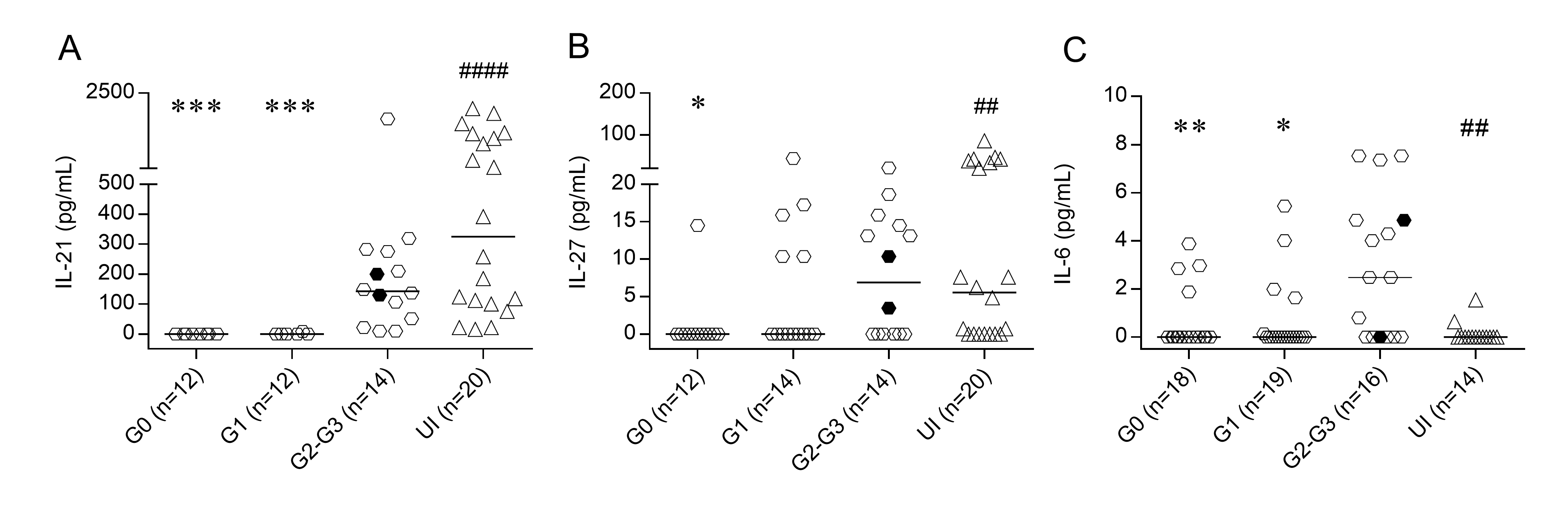

Supplement: S5 Fig — IL-21 and IL-27 were measured using ELISA, and IL-6 levels were measured using CBA. Each point represents the serum levels of IL-21 (A), IL-27 (B) and IL-6 (C) of individual subjects. Values under the limit of detection were graphed as zero. Horizontal lines indicate median values. Black symbols indicate subjects treated with benznidazole. Comparisons between clinical groups and uninfected subjects were performed using ANOVA followed by Dunn’s multiple comparison test. * p ≤ 0.05, ** p ≤ 0.01, *** p ≤ 0.001 compared with G2-G3. (A) ### p ≤ 0.001 compared with G0 and G1; (B) ## p ≤ 0.01 compared with G0; (C) ## p ≤ 0.01 compared with G2-G3. (TIF) [file pntd.0006998.s005.tif]
