# Supplementary material for: Trypanosoma cruzi-specific IFN-γ-producing cells in chronic Chagas disease associate with a functional IL-7/IL-7R axis
Source: PLoS Negl Trop Dis. 2018 Dec 5;12(12):e0006998. doi: 10.1371/journal.pntd.0006998 (PMC6281225; doi:10.1371/journal.pntd.0006998)
Supplement: S1 Table — (PDF) [file pntd.0006998.s006.pdf]

S1 Table. Cytokines serum levels in chronic Chagas disease patients

Cytokine[A]	G0 [B]		G1		G2-G3		
	P	NP	P	NP	P	NP	UI
IL-1β (pg/mL)	ND	ND	ND	ND	ND	ND	ND
IL-9 (ng/mL)	ND	ND	0.10 (0 – 2.01)	0 (0 – 2.10)	0.07 (0 – 1.40)	0 (0 – 0.57)	ND
IL-10 (pg/mL)	ND	ND	ND	ND	ND	ND	ND
IL-12 (pg/mL)	ND	ND	ND	ND	ND	ND	ND
TNF-α (pg/mL)	ND	ND	ND	ND	0 (0 – 6.57)	ND	ND)

[A] Serum levels of IL-9 were measured by ELISA and IL-1β, IL-10, IL-12 and TNF-α by CBA.

[B] Values are expressed as medians (interquartile range). ND non-detected, limits of detection: ELISA, 0.1 ng/mL; CBA IL-1β 7,2 pg/mL; IL-10 3,3 pg/mL; IL-12 1,9 pg/mL; TNF-α 3,7 pg/mL
